# 5-Aminolevulinate improves metabolic recovery and cell survival of the liver following cold preservation

**DOI:** 10.7150/thno.69446

**Published:** 2022-03-21

**Authors:** Xiaomei Zhang, Liang Chen, Wei Liu, Juan Shen, Haobin Sun, Jinliang Liang, Guo Lv, Guihua Chen, Yang Yang, Jingxing Ou

**Affiliations:** 1 Department of Hepatic Surgery and Liver transplantation Center of the Third Affiliated Hospital, Organ Transplantation Institute, Sun Yat-sen University; Organ Transplantation Research Center of Guangdong Province, Guangdong province engineering laboratory for transplantation medicine. Guangzhou 510630, China; 2 Guangdong Provincial Key Laboratory of Liver Disease Research, Guangzhou, China; 3 Key Laboratory of Liver disease biotherapy and Translational Medicine of Guangdong Higher Education Institutes, Guangzhou, China.

**Keywords:** Hibernator, Organ preservation, Hepatocyte-like cells, Mitochondria, 5-Aminolevulinate

## Abstract

**Rationale:** Hibernating thirteen-lined ground squirrels (GS; *Ictidomys tridecemlineatus*) are naturally adapted to prolonged periods of ultraprofound hypothermia (body temperature < 5 ºC) during torpor, and drastic oscillations of body temperature and ischemia/reperfusion-like stress during their short euthermic interbout arousals. Thus, their superior adaptability may hold tremendous promise for the advancement of donor organ cold preservation and subsequent organ transplantation. However, bridging hibernation research and translational medicine has been impeded by a dearth of *in vitro* research tools, till the recent establishment of the GS induced pluripotent stem cells (iPSCs). In this study, we reported the generation of functional hepatocyte-like cells (HLCs) from GS iPSCs. As temperature and oxygen supply affect cellular metabolism, we hypothesized that the GS HLCs can metabolically counter drastic temperature and oxygen supply changes. Differentially regulated metabolites can be evaluated and included into the preservation solution to mitigate temperature and ischemia/reperfusion-associated damage to donor livers.

**Methods:** A protocol has been developed to produce GS iPSCs-derived HLCs. Comparative metabolomic analysis on GS HLCs and human donor liver samples revealed changes in metabolites caused by cold storage and rewarming. Human embryonic stem cell (ESC)-derived HLCs and *ex vivo* cold preservation and reperfusion of isolated rat livers were used to assess candidate metabolites that may have protective effects against preservation-related injuries.

**Results:** GS iPSCs were efficiently differentiated into expandable, cryopreservation-compatible and functional HLCs. Metabolomic analysis unveiled distinct changes of mitochondrial metabolites between GS and human cells following cold storage and rewarming. GS and human HLC-based experiments indicated that the metabolism of 5-aminolevulinate (5-ALA) is key to restricting free radical production during rewarming. Survival of human HLCs was significantly increased following cold exposure and rewarming, as supplemented 5-ALA enhanced Complex III activity and improved mitochondrial respiration. Further, 5-ALA mitigated damage in rat livers following 48-h cold preservation and *ex vivo* reperfusion. Metabolomic and transcriptomic analyses revealed that supplemented 5-ALA promoted both anabolic and catabolic activities while alleviating cell death, inflammation, hypoxia and other stress responses in isolated perfused rat livers.

**Conclusion:** In the liver, rewarming from ultraprofound hypothermia imposes complex metabolic challenges and stresses on the mitochondria. Metabolites such as 5-ALA can help alleviate mitochondrial stress. Supplementing 5-ALA to the liver preservation solution can substantially improve the functional recovery of rat livers following prolonged cold preservation, rewarming and reperfusion.

## Introduction

In the majority cases of end-stage liver diseases, liver transplantation is the only practical option remaining. Static cold storage of donor livers in the University of Wisconsin (UW) solution has been the standard clinical practice for decades due to its cost efficiency and reliability 1-3. However, cold storage-associated injuries 4, and warm ischemia/reperfusion (IR) injury 5-7 occurred during organ procurement and transplantation surgery have restricted the cold preservation time for donor livers to typically no more than 12 h. In addition, these injuries were even more pronounced in livers from so-called 'marginal' donors 8, which account for an increasing proportion in the global liver donor pool. Such allografts are known to have a significantly higher rate of delayed graft function 9-13. Thus, resolving the challenge of liver cold and IR injuries would extend liver preservation time, improve patient outcomes and also expand the donor pool 14, 15. To this end, animal models acclimated to conditions similar to organ preservation and transplantation would offer the opportunity to systematically understand the biology of adaptation to drastic temperature fluctuations and warm IR.

Researchers have well demonstrated that hibernating ground squirrels can play such a role. These small mammals maintain their body temperature at 37 ºC in non-hibernating seasons; during hibernation, they live through cycles of deep torpor that lasts for days with a body temperature typically below 5 ºC, and short interbout arousal in which the animals quickly raise their body temperature to 37 ºC for a few hours while enduring IR-like physiology 16-18. It has been shown that livers from hibernating thirteen-lined ground squirrels (GSs; *Ictidomys tridecemlineatus*) are more resistant to both cold and warm IR injuries 19, 20. Neuroprotective effects have also been extensively studied in hibernating ground squirrel species, piquing interests in translating these spectacular feats into effective treatments of stroke and other IR-type of injuries to the brain 21-24.

Rewarming and IR simultaneously occur during liver transplantation surgery and in the transition between deep torpor and euthermic interbout arousal in hibernating GS, hence similar physiological responses may be evoked towards rewarming and IR. In warm IR, mitochondria overproduce reactive oxygen species (ROS), which have been proposed to arise from reverse electron transport (RET) 25-28: normally, mitochondrial respiratory Complexes I, III and IV pump protons across the inner mitochondrial membrane to maintain a mitochondrial membrane potential (Δψm), and Complex V uses the proton gradient to produce ATP; during IR, altered activities of Complex II (succinate dehydrogenase) and other enzymes such as dihydroorotate dehydrogenase (DHODH) cause over-reduction of coenzyme Q, which transports the excessive electrons back to Complex I, resulting in NAD^+^-NADH reduction and ROS overproduction; other outcomes in the IR-induced RET model include Δψm hyperpolarization and diminished mitochondrial ATP production. In human iPSC-derived neuronal cultures, cold exposure results in hyperpolarization of Δψm and ROS overproduction 29. Moreover, ROS overproduction has also been implied in aggravating organ damage upon rewarming; in stark contrast, hibernating GSs are capable of maintaining mitochondrial homeostasis, thus restricting ROS production and preventing cell death 30, 31. Despite their immense potential, not many labs are able to accommodate hibernating ground squirrel species as research models, while few hibernator-derived cell lines were available 32 to support studies on cold and IR adaptation at the cellular and molecular levels.

Recently, we established the first GS-derived induced pluripotent stem cell (iPSC) 29, 33, which may be differentiated into various cell types of interest. Here, we report an efficient method to produce high-quality GS iPSC-derived hepatocyte-like cells (HLCs), and the identification of 5-aminolevulinate (5-ALA) and other mitochondrial metabolites that were differentially regulated in GS HLCs following change of temperature or oxygen supply. As the precursor for heme production 34, 5-ALA has been proposed to enhance the activity of mitochondrial Complex IV (cytochrome c oxidase) in the mouse liver 35, protect cells from heat shock 36, and together with ferrous iron induce permanent cardiac allograft acceptance in mice 37. Here, we found that addition of 5-ALA in the cold preservation solution antagonized mitochondrial stress in human HLCs following cold exposure and rewarming, mitigated damage and facilitated metabolic recovery in isolated perfused rat livers after prolonged cold storage.

## Results

### GS iPSCs differentiation into functional HLCs

**Figure 1:**
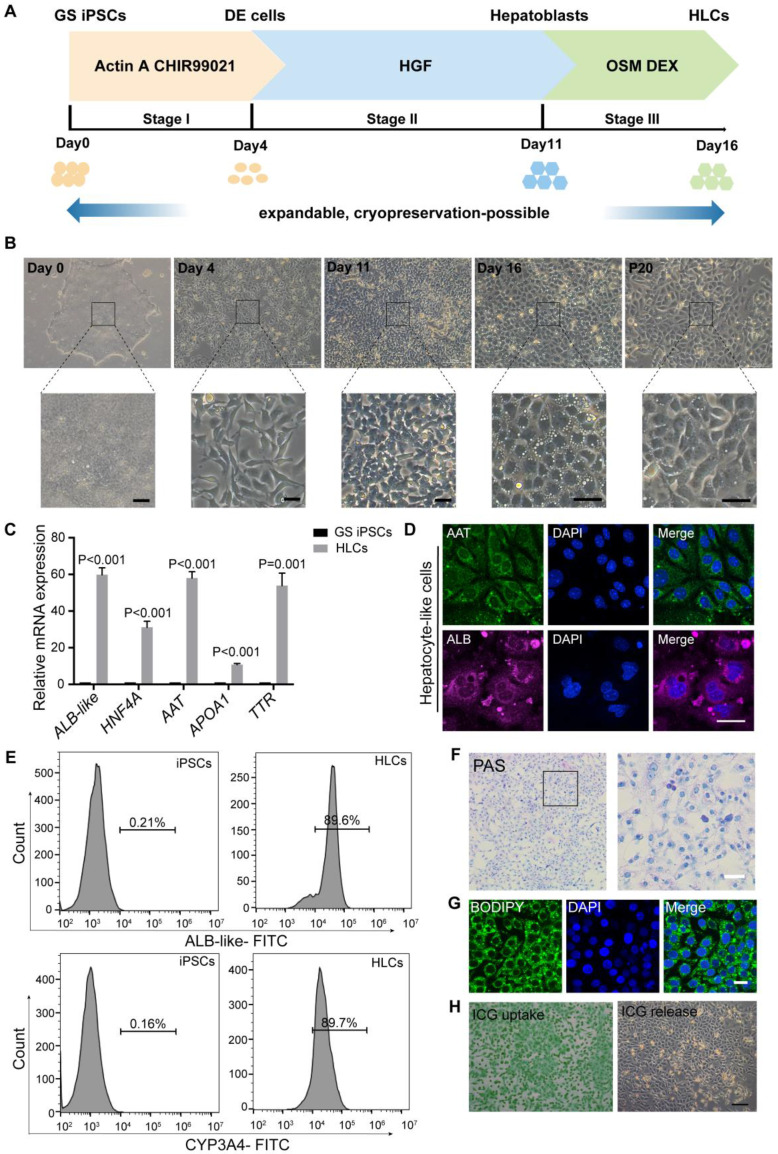
** Differentiation of ground squirrel induced pluripotent stem cells (GS iPSCs) into functional hepatocyte-like cells (HLCs). (A)** Schematic diagram of the three-stage protocol of GS iPSCs. **(B)** Representative images showing cellular morphological changes during the differentiation process; scale bars: 50 μm. **(C)** RT qPCR analysis of hepatocyte markers at the endpoint of stage III. GS iPSCs were used as control. Gene expression was normalized to that of *ACTB*. **(D)** Immunofluorescence staining of AAT and GS ALB-like proteins at the endpoint of stage III (n = 5 experiments); scale bar: 20 μm. **(E)** Percentage of ALB-like- and CYP3A4-positive cells of GS iPSCs and HLCs analyzed by flow cytometry (n = 3). **(F)** PAS staining showing glycogen storage in HLCs (n = 5); scale bar: 50 μm. **(G)** BODIPY staining indicating lipid storage in HLCs (n = 5); scale bar: 20 μm. **(H)** ICG uptake (left) and overnight-release (right) in HLCs (n = 5); scale bar: 200 μm. Data are expressed as mean ± SEM; Student's *t*-test for comparisons between iPSCs and HLCs; *P* values are indicated.

We first established a robust procedure to efficiently differentiate GS iPSCs into HLCs. Adapted from human pluripotent stem cell-based protocols 38-40, our three-stage differentiation procedure (Figure 1A-B; see **Methods** for details) resulted in up to 90% of the cells co-expressing markers for definitive endoderm (DE: SOX17 and GATA4) (Figure S1B), hepatoblasts (HNF4A and AFP) (Figure S1D) and HLCs (AAT and ALB) (Figure 1D).

To initiate the differentiation of GS iPSCs into DE cells, the cells were treated with CHIR99021, a highly selective GSK-3β inhibitor, and activin A, a member of the TGFβ family that stimulates WNT signaling. After four days of treatment, the mRNA levels of pluripotency markers *OCT4* and *NANOG* were downregulated (Figure S1A), expression of DE markers *SOXO17*, *GATA4* and *FOXA2* was substantially enhanced (Figure S1A), whereas the morphology of stem cell colonies had altered from a dense cluster to a petal-like shape (Figure 1B). Subsequent formation of hepatoblasts was achieved by 7-day treatment with HGF, during which the cells transformed into a cuboid shape (Figure 1B) and expressed high levels of hepatoblast markers *HNF4*, *AFP*, *CK18*, *CK19* and *TBX3* (Figure S1C). For further differentiation into HLCs, the cells were treated with Oncostatin M, an interleukin 6-related cytokine, and dexamethasone for 5 days. Hereafter, the differentiated GS HLCs developed a cobblestone shape with multiple cytoplasmic vacuoles and vesicles, and conspicuous nucleoli, while some cells were binucleated (Figure 1B). The GS HLCs highly expressed hepatocyte markers *HNF4A*, *AAT*, *TTR*, *APOA1* (Figure 1C), GS albumin (ALB)-like proteins, and cytochrome P450 (CYP) isoenzymes 41 (Figure 1E and Figure S2). The elevated expression and secretion of ALB-like proteins, and declined expression of AFP (Figure S1E-F) suggested hepatocyte maturation 42. The differentiating GS cells remained proliferative and could be cryopreserved, while GS HLCs can be further passaged for up to 20 passages. Hence our method can prepare GS HLCs for Omics and other assays that require cells in large quantities.

The GS iPSC-derived HLCs were subjected to further evaluations. Hepatocytes synthesize and store glycogen, which can be assessed by Periodic acid-Schiff (PAS) staining 40. Functional GS HLCs were stained pink to purple in PAS staining, indicating glycogen synthesis (Figure 1F). Another hepatocyte feature was shown via BODIPY staining to reveal lipid droplets in GS HLCs (Figure 1G). Indocyanine green (ICG) is a dye that can be ingested and excreted by the liver and hence is clinically used to assess liver functions. As expected, GS HLCs could uptake and release ICG (Figure 1H). These hepatic functional assessments presented warrants for the quality of GS iPSC-derived HLCs generated with our method.

### Metabolomic profiles highlighting metabolic responses to temperature and oxygen changes in GS HLCs

When temperature or oxygen supplies changes, cellular metabolism is among the first biological activities to be affected. Hibernators may have evolved to put up their first adaptative response to these changes at the metabolic level, which is difficult to analyze via *in vivo* experiments. In the case of donor liver cold storage and transplantation, it is impractical to discern the impact of rewarming from that of IR during the transplantation of donor livers. However, since tens of millions of GS iPSC-derived HLCs can be generated at a time, *in vitro* cold exposure, rewarming, hypoxia and reoxygenation experiments could be performed. Then, the impact of temperature or oxygen change on GS HLC metabolome can be analyzed and compared to human donor liver samples from cold storage and post-transplantation (see **Methods**).

**Figure 2:**
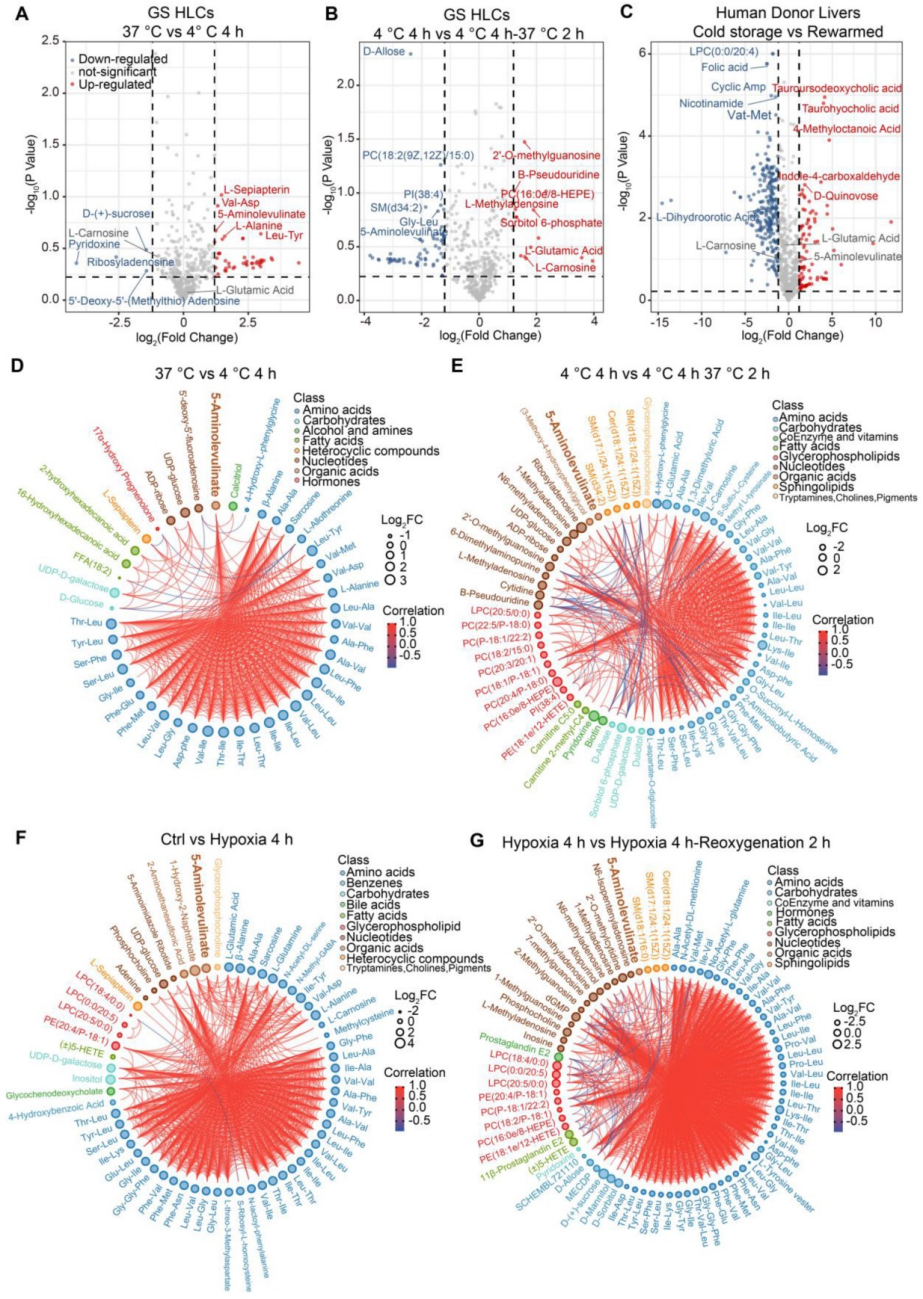
** Metabolomic profiling of differentially regulated metabolites in GS HLCs subjected to temperature or oxygen supply change.** Volcano plot of metabolites in GS HLCs at 37 °C versus 4 °C 4 h **(A)** up-regulated metabolites at 4 °C 4 h, red; down-regulated metabolites, blue) and 4°C 4 h versus 4 °C 4 h 37 °C 2 h **(B)** up-regulated metabolites at 4 °C 4 h 37 °C 2 h, red; down-regulated metabolites, blue); n = 3 culture replicates for each group. Also see Data S1. **(C)** Volcano plot analysis of differential metabolites in human donor livers during cold storage versus post-transplantation (rewarmed and perfused) group (n = 10 donors). Also see Data S2. **(D)** Chord plot depicting associations between metabolites in GS HLCs at 37 °C versus 4 °C 4 h; n = 3 culture replicates for each group. Metabolites are arranged by metabolite classes. The size of outer circle of each metabolite represents its Log_2_FC value. The inner red line indicates positive correlation and blue line indicates negative correlation. **(E)** Chord plot depicting associations between metabolites in GS HLCs at 4 °C 4 h versus 4 °C 4 h 37 °C 2 h; n = 3 culture replicates for each group. **(F)** Chord plot depicting associations between metabolites in GS HLCs of control versus hypoxia 4 h; n = 3 culture replicates for each group. **(G)** Chord plot depicting associations between metabolites in GS HLCs of hypoxia 4 h versus hypoxia 4 h reoxygenation 2 h; n = 3 culture replicates for each group. Also see Data S3.

Apparently, small peptides, amino acids and their derivatives were similarly expended in both rewarmed GS HLCs and post-transplantation donor liver samples (Figure S3, Data S1 and S2), suggesting that during such transition small peptide and amino acid metabolism may be elevated in both GS and human hepatocytes. Among the differentially regulated metabolites in GS HLCs during cold exposure and rewarming (Figure 2A-B and Data S1), we noted that 5-aminolevulinate (5-ALA) was up-regulated at 4 °C and down-regulated following rewarming, whereas L-Carnosine and L-Glutamic acid were up-regulated following rewarming. These metabolites are associated with mitochondrial metabolism (Figure S4A-C). In contrast, the levels of these mitochondrial metabolites were not altered in human donor livers transiting from cold storage to the post-transplantation stage (Figure 2C and Data S2), during which the livers endure stress from rewarming and warm IR.

Comparing the metabolomic profiles of GS HLCs subjected to hypoxia (1% O_2_) or hypoxia-reoxygenation transition to those of GS HLCs subjected to 4 °C storage or cold-rewarming transition may unveil metabolic regulations common to the two types of transitions. Notably, 5-ALA appeared to accumulate in both cold and hypoxic conditions, and then dissipate after recovery, whereas the level of L-Glutamic acid remained up-regulated in hypoxic and reoxygenated conditions, and L-Carnosine showed a trend of down-regulation during hypoxia (Figure 2D-G, Figure S4D, Data S1 and S3).

### Cellular redox homeostasis and energy metabolism protected by 5-ALA in rewarmed human HLCs

**Figure 3:**
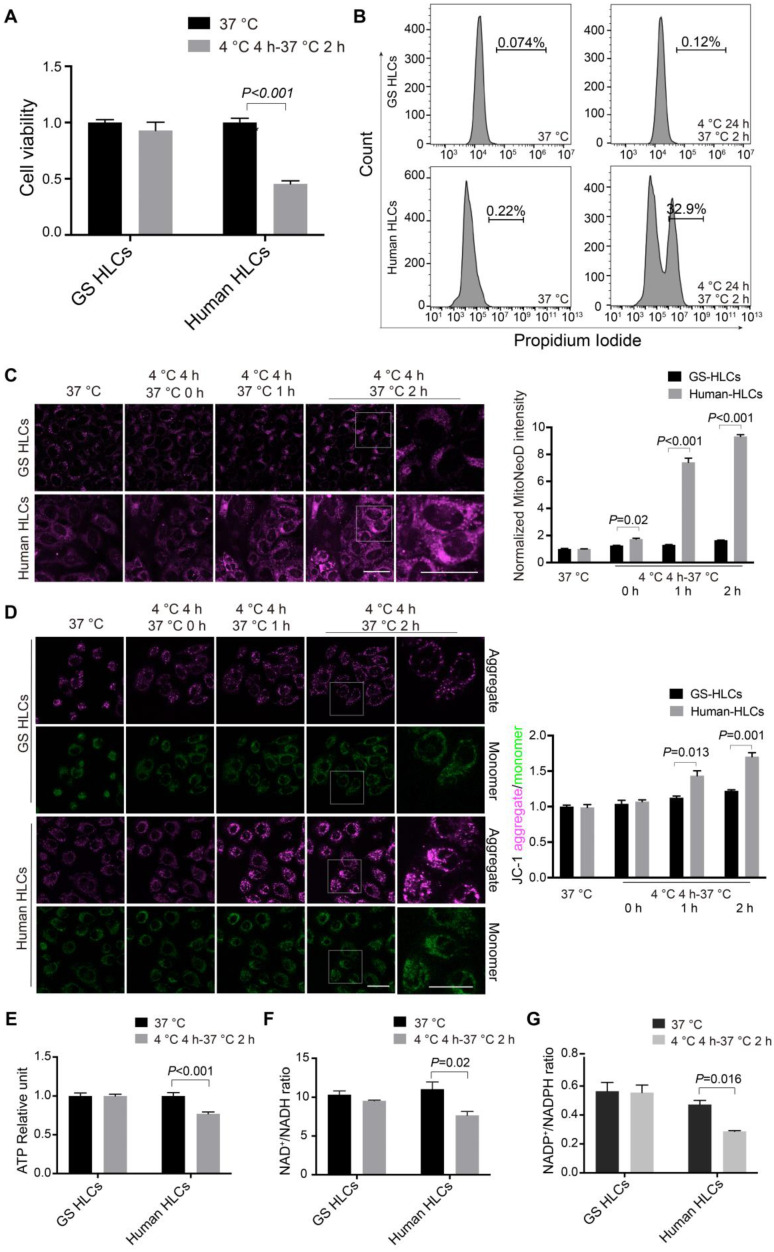
** Rewarming-aggravated cell death and metabolic dysfunction in human HLCs.** Viability of GS and human HLCs following 24-h cold exposure and then 2-h rewarming, measured by CCK8 (**A**) and Propidium Iodide staining (**B**) (n = 5 experiments). (**C**) Left: live imaging of MitoNeoD to assess mitochondrial ROS during 2-h rewarming in GS and human HLCs; scale bars: 20 μm; Right: quantification of MitoNeoD fluorescence intensity (n = 5 experiments). (**D**) Left: live imaging of JC-1 aggregate- and monomer-fluorescence to assess mitochondrial membrane potential (Δψm) in GS and human HLCs during 2-h rewarming; scale bars: 20 μm; Right: quantification of JC-1 aggregate/monomer intensity (n = 5 experiments). ATP levels (**E**), and the ratios of NAD+/NADH (**F**) and NADP+/NADPH (**G**) measured in rewarmed GS and human HLCs (n = 5 experiments). Data are expressed as mean ± SEM; Student's *t*-test for comparisons between GS and human; *P* values are indicated.

As neuronal cultures from non-hibernating species are susceptible to cold-induced mitochondrial stress 29, here we first sought to test how well human HLCs can endure cold incubation and rewarming. Human embryonic stem cells (ESCs) were differentiated into high-quality, functional HLCs (Figure S5). Significant cell death was noted in human ESC-derived HLCs incubated at 4 °C for 24 h followed by 2-h incubation at 37 °C, but not in GS HLCs (Figure 3A-B and Figure S6). Then MitoNeoD, a highly specific dye for mitochondrial ROS production 43 was used in live HLC imaging. A modestly elevated ROS production during 4-h cold exposure and a drastic increase of mitochondrial ROS within the first 2 h of rewarming were found in human HLCs, but not in GS HLCs (Figure 3C).

In addition, fluorescent dye JC-1 was used to monitor Δψm: when Δψm increases (hyperpolarization), JC-1 aggregates accumulate and hence their fluorescent intensity (shown in magenta) increases; when Δψm decreases (depolarization), the monomer state of JC-1 is prompted and hence monomers' fluorescence (shown in green) intensifies. As expected, Δψm rapidly hyperpolarized within the first 2 h of rewarming in human HLCs, but not in GS HLCs (Figure 3D). Next, ATP production and the ratios of NAD^+^/NADH and NADP^+^/NADPH were all decreased in rewarmed human HLCs but not in GS HLCs (Figure 3E-G), indicative of poor metabolic recovery and plausible cause to cell death 44, 45 in human HLCs.

**Figure 4:**
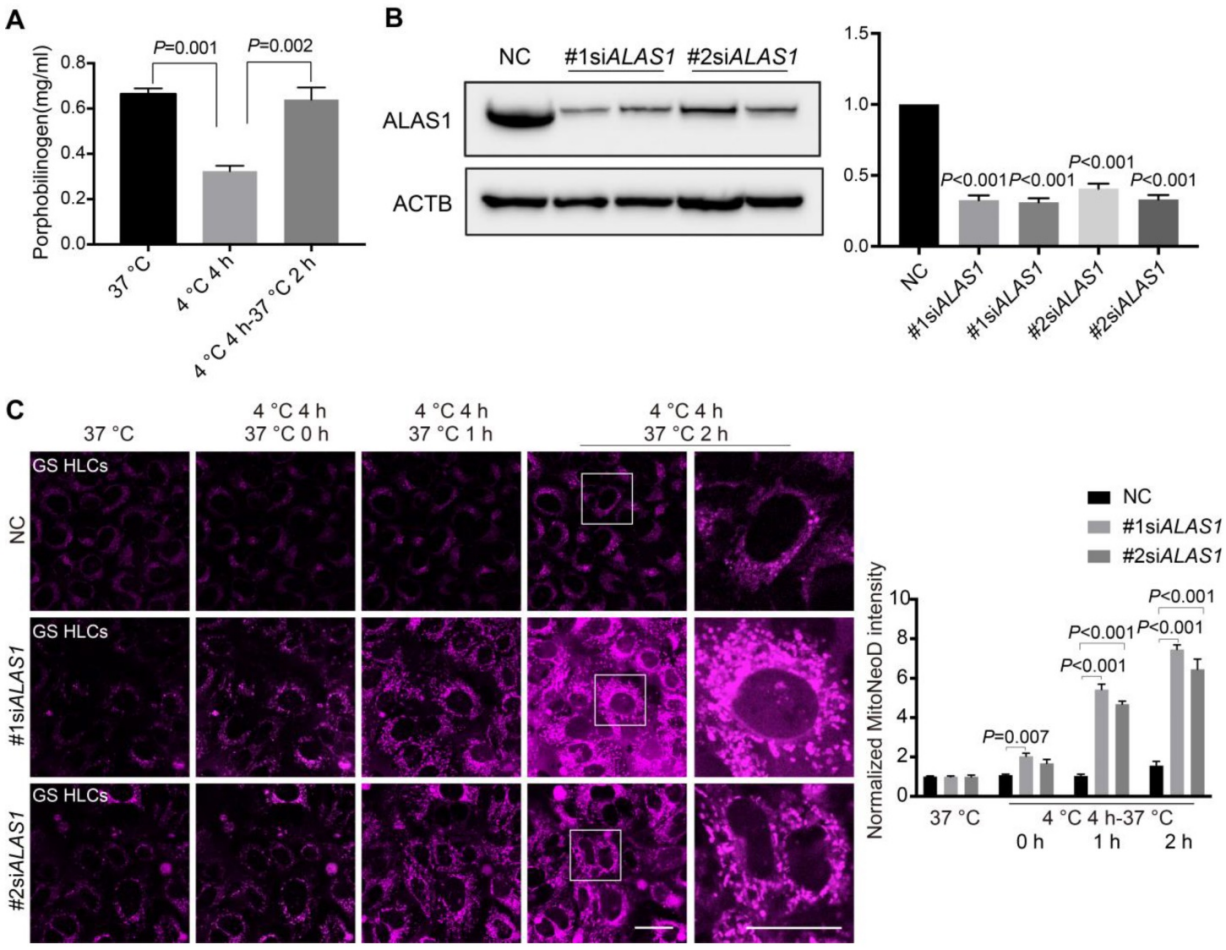
** 5-ALA metabolism and mitochondrial redox homeostasis in GS HLCs during temperature fluctuations.** (**A**) Measurement of porphobilinogen (PBG), the downstream metabolite of 5-ALA metabolic pathway (see Figure S4A), at indicated conditions in GS HLCs (n = 3 experiments). (**B**) Left: immunoblots of ALAS1 in control siRNA- and *ALAS1* siRNA-treated GS HLCs; Right: normalized intensity levels of ALAS1 protein (n = 3 experiments); NC: negative control. (**C**) Left: live imaging of MitoNeoD to evaluate mitochondrial ROS in GS HLCs at indicated conditions; scale bars: 20 μm; Right: normalized intensity levels of MitoNeoD (n = 3 experiments). Data are expressed as mean ± SEM; one-way ANOVA followed by Dunnett multiple-comparisons test versus 4 ºC 4 h (A) or NC (B-C); *P* values are indicated.

Because 5-ALA metabolism in GS HLCs appeared to be responsive to cold incubation and rewarming (Figure 2, Figure S4 and Data S1), 5-ALA synthesis is an important metabolic activity in hepatocytes leading to the production of porphobilinogen (PBG) and subsequently heme 34 (Figure S4A), and heme is essential to mitochondrial respiration 46 and CYP functions 47 that the liver metabolic activities heavily rely on, we then assessed whether 5-ALA metabolism supports cellular adaptation to cold and rewarming. Consistent with the alteration in 5-ALA levels, PBG levels in GS HLCs declined during cold exposure and recovered during rewarming (Figure 4A). Further, disruption of 5-ALA synthesis by knocking down 5-aminolevulinate synthase 1 (ALAS1) in GS HLCs resulted in substantial mitochondrial ROS overproduction during rewarming (Figure 4B-C), strongly suggesting that mitochondrial recovery during rewarming largely depends on 5-ALA metabolism in GS HLCs.

**Figure 5:**
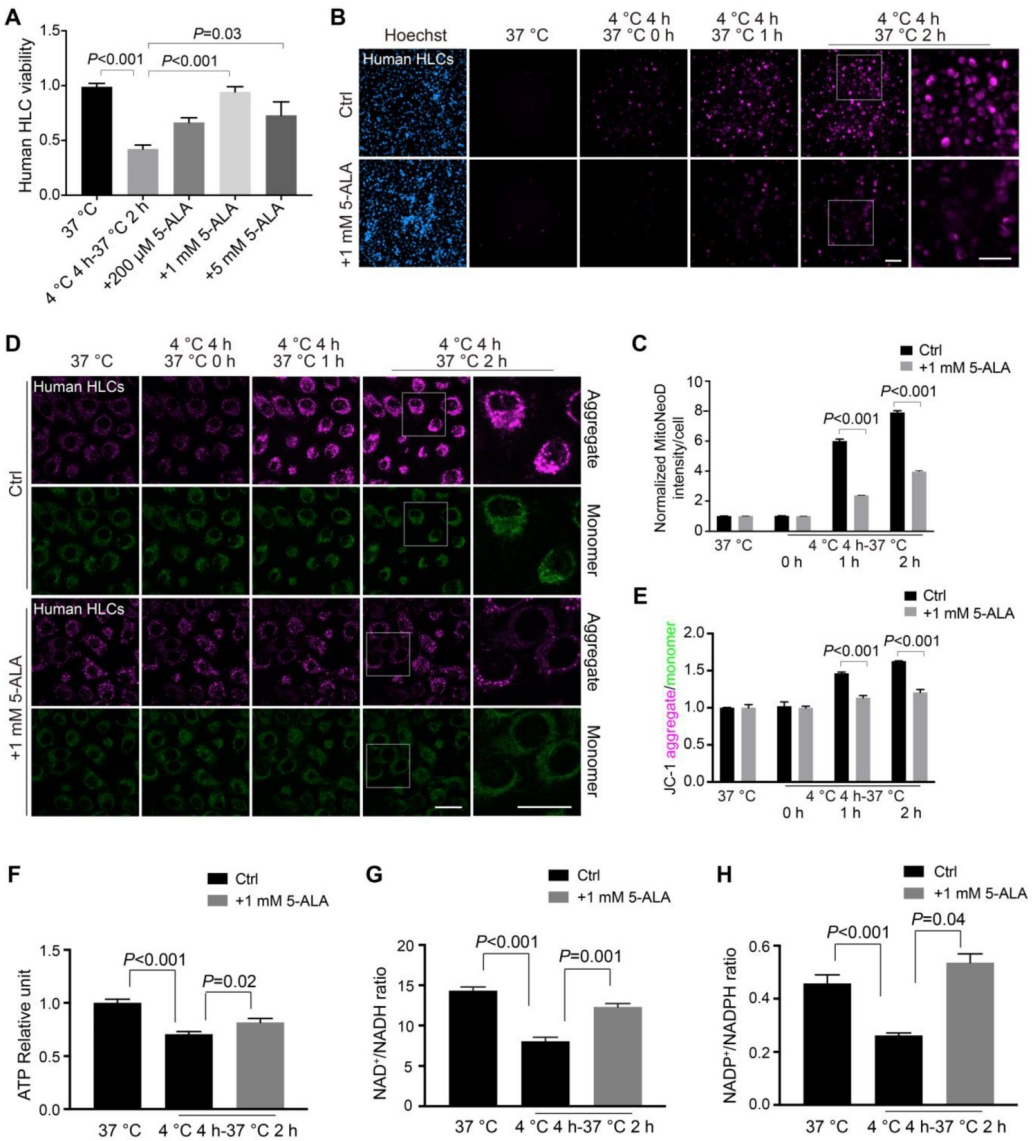
** Cellular survival and metabolic recovery in rewarmed human HLCs facilitated by 5-ALA.** (**A**) Cell viability in human HLCs at indicated conditions (n = 5 experiments). (**B**) Automatic high-content imaging of MitoNeoD fluorescence in human HLCs at indicated conditions; scale bars: 100 μm. (**C**) Quantitative analysis of MitoNeoD intensity per cell from (B) (n = 5 experiments). (**D**) Live imaging of JC-1 aggregate- and monomer-fluorescence in human HLCs at indicated conditions; scale bars: 20 μm. (**E**) Quantitative analysis of JC-1 aggregate/monomer intensity from (D) (n = 5 experiments). ATP levels (**F**), and the ratios of NAD+/NADH (**G**) and NADP+/NADPH (**H**) measured in human HLCs at indicated conditions (n = 5 experiments). Data are expressed as mean ± SEM; one-way ANOVA followed by Dunnett multiple-comparisons test versus 4 ºC 4 h-37 ºC 2 h (A) or Ctrl, 4 ºC 4 h-37 ºC 2 h (F-H); Student's *t*-test between two comparisons (C, E); *P* values are indicated.

Inspired by these findings, we then tested if 5-ALA supplement (Figure S7A) would antagonize rewarming-induced stresses in human HLCs. Because pre-treatment with 5-ALA only during the room-temperature incubation showed little effect on cellular energy homeostasis (Figure S7B-D), in this study 5-ALA was added prior to and during the 4 °C incubation, while no extra 5-ALA was provided in the rewarming process. In our hands, human HLCs incubated with 0.2-5 mM 5-ALA at 4 °C had improved cell viability following rewarming (Figure 5A); addition of 0.2-5 mM 5-ALA also effectively blocked mitochondrial ROS overproduction (Figure 5B-C and Figure S8A) and well maintained the Δψm (Figure 5D-E and Figure S8B); after rewarming, human HLCs incubated with 1 mM 5-ALA at 4 °C greatly recovered their ATP production and NAD^+^/NADH and NADP^+^/NADPH ratios (Figure 5F-H).

**Figure 6:**
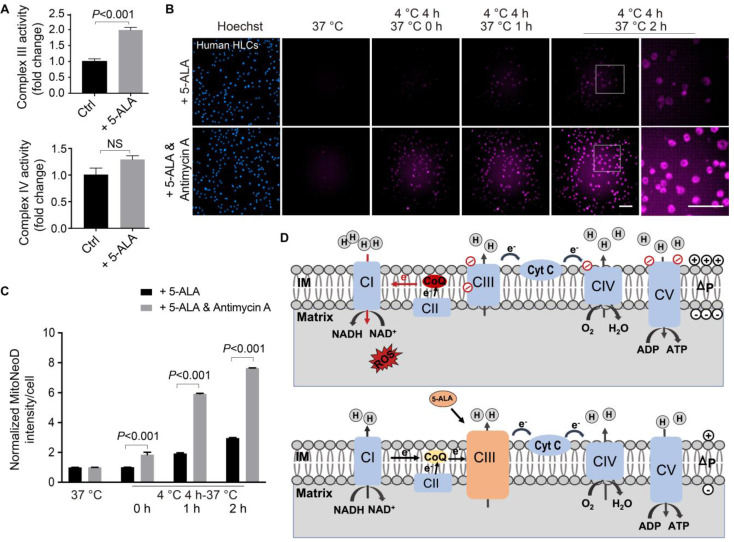
** 5-ALA action through Complex III during rewarming in human HLCs.** (**A**) Up: activity of Complex III measured in rewarmed human HLCs at indicated conditions; Down: activity of Complex IV measured in rewarmed human HLCs at indicated conditions (n = 5 experiments). (**B**) Automatic high-content imaging of MitoNeoD fluorescence in human HLCs at indicated conditions; scale bars: 100 μm. (**C**) Quantification of MitoNeoD fluorescence intensity from (B) (n = 5 experiments). (**D**) Schematic diagrams of a possible source of ROS overproduction from reverse electron transport (RET) (up) and the protective effect of 5-ALA via Complex III (down) during rewarming. Data are expressed as mean ± SEM; Student's *t*-test between two comparisons;* P* values are indicated; NS: not significant.

Since NAD^+^/NADH and NADP^+^/NADPH ratios and ATP production reflect the global cellular metabolic homeostasis, we next sought to pinpoint the mitochondrial targets modulated by the supplemented 5-ALA. Here in the 5-ALA-treatment group, we observed that rewarmed human HLCs had higher mitochondrial Complex III (coenzyme Q-cytochrome c reductase) activity, but not Complex IV (Figure 6A). If Complex III was simultaneously inhibited by antimycin A, then 5-ALA could no longer antagonize cold and rewarming-induced mitochondrial ROS overproduction (Figure 6B-C). As the DHODH-catalyzed dihydroorotate-orotate oxidation and coenzyme Q reduction contribute to IR-induced mitochondrial ROS production 28, 48, and dihydroorotate levels drastically decreased while orotate levels mildly increased in post-transplantation donor liver samples (Data S2), DHODH activity was tested in human HLCs. Indeed, DHODH activity was elevated in human HLCs following rewarming, which was barely affected by 5-ALA supplement (Figure S9).

Taken together, here we propose that rewarming-induced mitochondrial stress in human HLCs is similar to the IR-induced RET 25-28, which can be ameliorated by the 5-ALA-enhanced Complex III activity (Figure 6D; see details in **Discussion**).

### Mitigating cold storage and rewarming-induced damage in rat livers by 5-ALA

**Figure 7:**
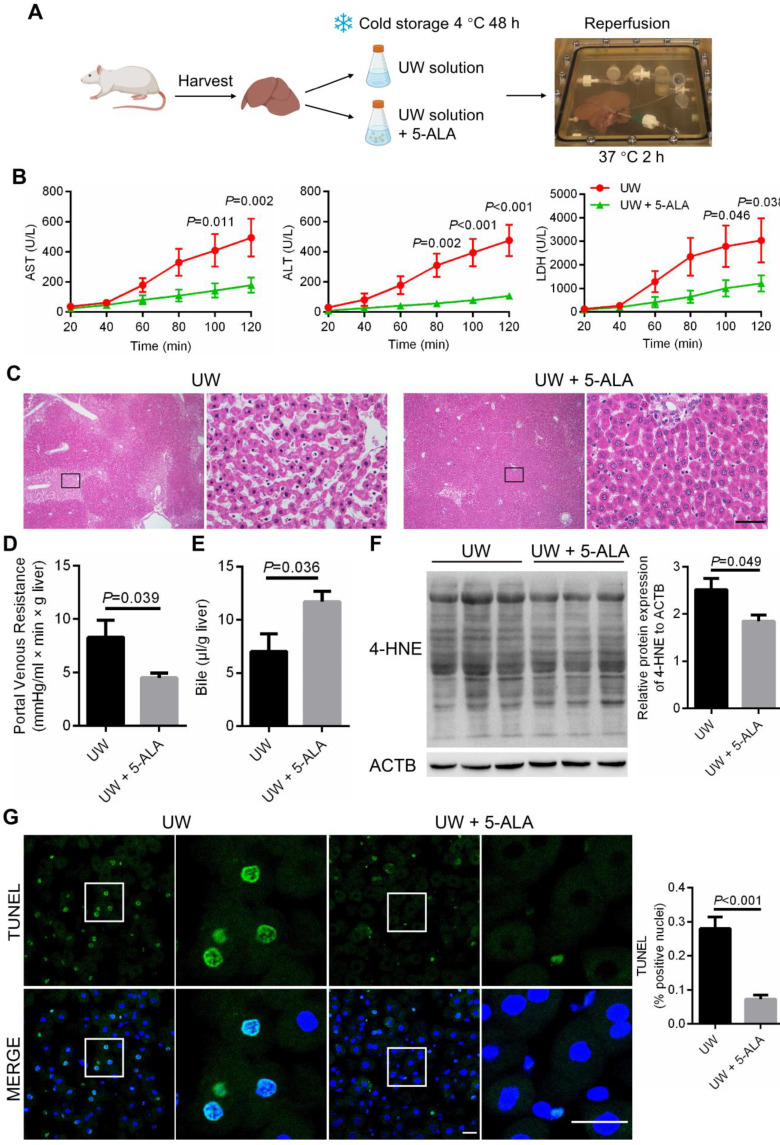
** Cold preservation, rewarming and reperfusion-induced injuries ameliorated by 5-ALA in an isolated perfused rat liver model. (A)** Schematic diagram showing the procedure for cold storage, rewarming and reperfusion of isolated rat livers. **(B)** The levels of aspartate aminotransferase (AST), alanine aminotransferase (ALT) and lactate dehydrogenase (LDH) in perfusate samples from isolated rat livers at indicated conditions; samples were collected every 20 min during perfusion (n = 6 animals per group). **(C)** Hematoxylin-eosin staining of rat liver sections; scale bar: 50 μm. Portal vein resistance **(D)** and bile production **(E)** in isolated rat livers at indicated conditions after 48-h storage at 4 ºC followed by 2-h normothermic reperfusion (n = 6 samples per group). **(F)** Left: immunoblots of proteins modified by 4-hydroxynonenal (4-HNE) from protein extracts of isolated rat livers after 2-h normothermic reperfusion; Right: normalized 4-HNE intensity levels (n = 3 experiments). **(G)** Left: TUNEL staining of rat liver sections at indicated conditions after 48-h storage at 4 ºC followed by 2-h normothermic reperfusion; scale bars: 20 μm; Right: percentage of TUNEL-positive apoptotic cells (n = 5 experiments). Data are expressed as mean ± SEM; Student's *t*-test between two comparisons;* P* values are indicated.

We next validated the protective role of 5-ALA using isolated perfused rat liver 49, a classic model to measure liver injuries inflicted by preservation and IR (Figure 7A; also see **Methods**). Following 48-h storage at 4 °C in UW solution with the addition of 5-ALA, perfusate samples from rat livers during normothermic perfusion contained significantly lower levels of aspartate aminotransferase (AST), alanine aminotransferase (ALT) and lactate dehydrogenase (LDH) compared to that from UW solution control group (Figure 7B), indicative of less liver damage in the 5-ALA group. Better preserved liver morphology and function were also revealed by normal sinusoidal space, increased bile production and decreased portal venous resistance in livers of the 5-ALA group (Figure 7C-E). The intensity of 4-HNE immunoblotting, a signature of lipid peroxidation, was milder in liver samples from the 5-ALA group than that from the control group (Figure 7F). TUNEL staining supported the notion that rat livers stored in UW solution with 5-ALA had fewer apoptotic cells than the control group (Figure 7G).

**Figure 8:**
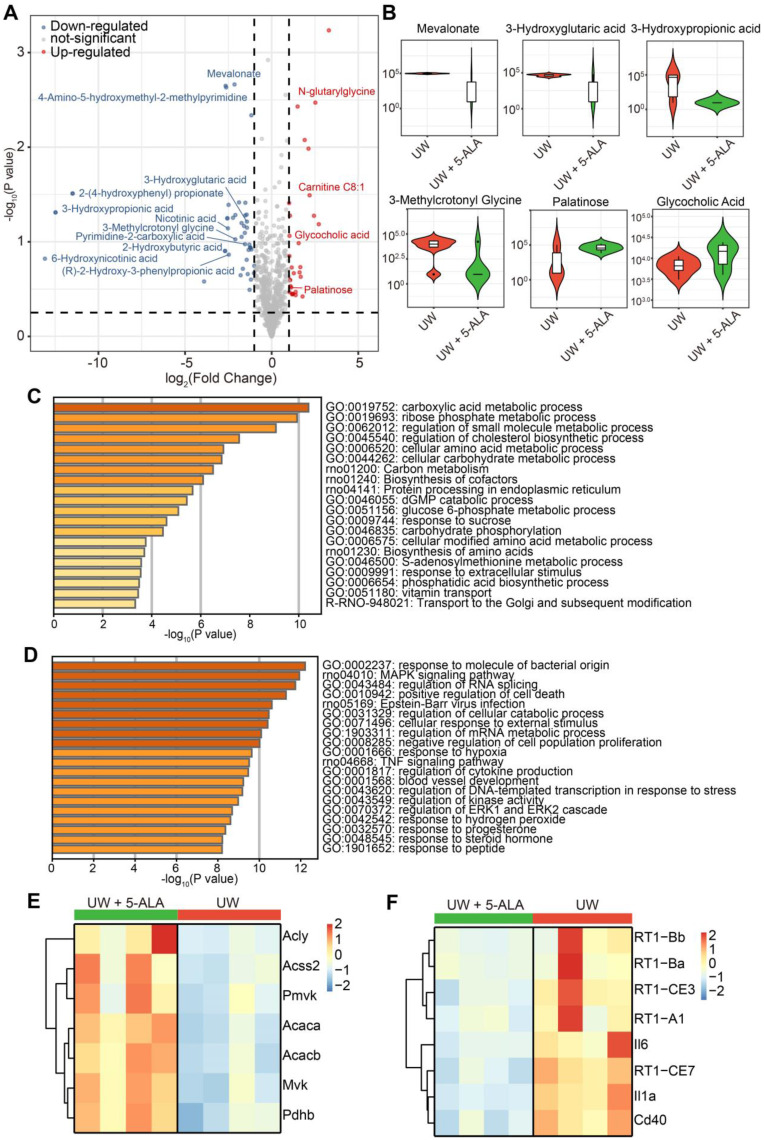
** Metabolomic and transcriptomic assessments on the effects of 5-ALA treatment in isolated perfused rat livers.** (**A**) Volcano plot of differential metabolites in isolated perfused rat livers following 48-h cold storage in UW solution versus UW solution supplemented with 5-ALA (up-regulated metabolites in UW + 5-ALA group, red; down-regulated metabolites, blue); n = 6 liver samples each group. (**B**) Violin Plots showing the abundance of some differential metabolites from (A). Also see Data S4. (**C**) Pathway analysis on transcripts up-regulated in rat livers of the UW + 5-ALA group; n = 4 liver samples each group. (**D**) Pathway analysis on transcripts up-regulated in rat livers of the UW group; n = 4 liver samples each group. (**E**) Heatmap showing the expression levels of selected genes that are involved in the acetyl-CoA and acyl-CoA metabolic pathways (see Data S5, pathway IDs GO:0006084 and GO:0006637). (**F**) Heatmap showing the expression levels of selected genes indicative of allograft failure (see Data S5, pathway IDs rno05332 and rno05330).

Further, metabolomic analysis indicated that compared to those in the control group, isolated perfused rat livers treated with 5-ALA significantly down-regulated the levels of metabolites such as 3-Hydroxypropionate, 3-Hydroxyglutarate, 3-Methylcrotonyl Glycine and mevalonate, and up-regulated the levels of metabolites such as N-Glutarylglycine and palatinose (Figure 8A-B and Data S4). High levels of 3-hydroxypropionate and related propionate compounds have been suggested to disturb acetyl-CoA oxidation, cripple mitochondrial ATP production and may be toxic to the transplanted liver 50. Interestingly, N-Glutarylglycine and 3-Hydroxyglutarate have been suggested to be downstream of glutaryl-CoA metabolism 51, while 3-Hydroxyglutarate accumulation has been implied to act as a mitochondrial uncoupler and impair brain energy metabolism in rats 52. Likewise, accumulation of 3-Methylcrotonyl Glycine has been suggested to compromise mitochondrial energy homeostasis and exacerbate lipid peroxidation in rat brains 53. The mevalonate pathway is crucial to cholesterol synthesis, while the blockade of mevalonate catabolism has been shown to depolarize Δψm and increase cell apoptosis 54. Correspondingly, palatinose has been suggested be beneficial to hepatic lipogenesis and cholesterol homeostasis 55.

Consistently, transcriptomic pathway analysis revealed that isolated perfused rat livers in the 5-ALA group comprehensively enhanced their catabolic and anabolic activities; in stark contrast, those in the control group had elevated stress responses to cell death and hypoxia, and higher expression of genes indicative of allograft failure (Figure 8C-F and Data S5). Overall, addition of 5-ALA into the UW solution profoundly ameliorated metabolic activities and mitigated injuries caused by cold preservation, hypoxia, rewarming and reperfusion in isolated perfused rat livers.

## Discussion

Donor tissue and organ static cold preservation remains to be the most practical option prior to transplantation, yet not much technological advancement has been made in the past 3 decades, thus limiting the preservation time and surgery outcomes. With the pluripotent stem cell-derived HLCs from GS and human, we were able to directly evaluate how the same temperature-switching process may differentially affect GS and human cellular physiology and survival. Here we propose that 5-ALA metabolism is crucial to the recovery of mitochondrial respiration and maintenance of redox homeostasis in HLCs during rewarming. Further, a simple supplementation of 5-ALA into the liver preservation solution managed to mitigate rewarming and reperfusion-induced injuries to isolated perfused rat livers. With these stem cell-based HLC platforms, other novel strategies may be inspired, tested and optimized to renovate the clinical practice for donor liver cold storage, transplantation and post-surgery care.

### Features of GS iPSC-HLCs

High-quality, functional GS HLCs can be differentiated from iPSCs by a protocol similar to that used in deriving human ESCs into HLCs (see **Methods**). In our hands, a distinct GS-specific feature is that the differentiating cells retain their proliferative potential longer than human cells and can be cryopreserved for future use. Despite that the GS ALB has not been well characterized, here with qPCR and an anti-human ALB antibody (see **Methods**) we confirmed the expression and secretion of GS ALB-like proteins in GS iPSC-HLCs (Figure 1C-E and Figure S1E-F). Further, it would be interesting to make GS iPSC-HLCs into 3-dimensional liver organoids in order to obtain more mature GS hepatocytes.

The GS HLCs can serve as a model intrinsically adapted to drastic temperature and oxygen supply changes. Rewarming-induced mitochondrial dysfunction is likely a primary contributor to poor recovery and cell death in human HLCs (Figure 3 and Figure S6). In contrast, GS HLCs appeared to exploit 5-ALA metabolism (Figure 2-4, Figure S4 and Data S1) to maintain mitochondrial homeostasis. Meanwhile, other metabolic activities that involve L-Glutamic acid and L-Carnosine may also play a role in HLC recovery. Note that tissue-specific regulations on mitochondrial activities are observed in hibernators 56, 57. With the GS iPSCs, different cell types can be derived to assess the biological significance of 5-ALA metabolism and others in the context of adaptation to and recovery from temperature and oxygen supply changes.

### Liver metabolic adaptation to the rewarming process

Hibernators are masters of metabolic adaptation. Integrative transcriptomic and metabolomic analyses suggested that the GS retina shifts its fuel use from carbohydrates to lipids and amino acids during hibernation 58. From the metabolomic profiles of GS HLCs and human donor livers (Figure 2, Figure S3-S4, and Data S1-S2), many small peptides, amino acids, and glycerophospholipids were down-regulated following rewarming in GS and human, implying that these metabolic pathways are active in the rewarming process. Meanwhile, the levels of 5-ALA, L-Glutamic acid and L-Carnosine were altered in GS but not in human, suggesting that certain mitochondrial metabolic activities may be differentially regulated between the two species. Understanding how mitochondrial homeostasis is maintained during drastic changes of temperature or oxygen supply is pivotal to the advance of tissue/organ cold preservation and post-transplantation care.

Here we discovered that the 5-ALA metabolic pathway is crucial to the recovery of normal hepatic functions from cold stress in GS, rat and human. Consistent with previous observation in human iPSC-neuronal cultures 29, cold exposure of human ESC-HLCs led to increased mitochondrial ROS production, the magnitude of which was further aggravated with Δψm hyperpolarization during rewarming (Figure 3C-D). While the levels of PBG, a metabolite downstream of 5-ALA synthesis, increased in GS HLCs following rewarming (Figure 4A), disrupting 5-ALA synthesis by knocking down *ALAS1* led to severe rewarming-induced mitochondrial ROS overproduction (Figure 4B-C). Contrarily, addition of 5-ALA into the cold-incubation medium was sufficient to mitigate rewarming-triggered mitochondrial dysfunction and cell death in human HLCs (Figure 5 and Figure S8). Because 3-Hydroxyglutarate and mevalonate may act as mitochondrial uncouplers at the expense of cellular energy homeostasis and survival 52, 54, the accumulation of these potentially harmful metabolites in isolated perfused rat livers (Figure 8A-B and Data S4) may be a response to cold and rewarming-induced Δψm hyperpolarization, enhanced 5-ALA metabolism might liberate the mitochondria from these extreme measures, hence facilitating the recovery of various metabolic activities in the liver (Figure 8C-F).

Among the complex roles 5-ALA plays in liver metabolic adaptation to the rewarming and reperfusion processes, we propose that 5-ALA contributes to mitochondrial functional recovery by enhancing Complex III activity (Figure 6). In animals, 5-ALA serves as the first compound in the porphyrin-heme synthesis pathway 34, while heme is integrated with Complex II, Complex III and Complex IV of the mitochondrial electron transport chain 46. In warm IR, RET has been suggested as the cause of mitochondrial stress 25-28, as over-reduction of coenzyme Q driven by the activities of Complex II and/or DHODH may overwhelm the activity of Complex III and hence relay the excessive electrons back to Complex I. Correspondingly, here we discovered that DHODH activity was augmented in human HLCs during the rewarming process (Figure S9A), which could contribute to RET-like mitochondrial stress and hence poor liver functions and cell death. Facilitated by 5-ALA metabolism, Complex III may accommodate the extra electron loads driven by Complex II and/or DHODH, and hence the mitochondria are able to gear up for the high metabolic demands of the recovering liver.

## Methods

### Human liver samples

Human liver samples for metabolomic analysis were obtained from deceased donors who give consents to research. The use of human tissue samples complied with the Declaration of Helsinki and was approved by Institutional Ethics Committee of the Third Affiliated Hospital of Sun Yat-Sen University. Livers in cold storage (CS) group (n = 10 per group) were collected when the donated liver were cold-stored for 4-6 h in UW solution (Organ Recovery Systems, USA) at 4 °C during trimming. Livers in the rewarmed group were matched samples collected 2 h during liver transplantation. Baseline characteristics of patients were collected in an anonymized fashion (Table S1).

### Cell culture

For stem cell cultures, Matrigel (Corning, see Table S2) was diluted to 4% with F12 medium (Stem Cell Technologies). Culture dishes (Falcon) were coated with 4% Matrigel overnight at 4 °C.

The human ESC line H1 (WA01) was originally purchased from WiCell Research Institute (Madison, WI) and maintained in the Third Affiliated Hospital of Sun Yat-sen University. All experiments involving the use of the H1 ESC line were performed in accordance with the guidelines and regulations of the Research Ethics Committee at the Third Affiliated Hospital of Sun Yat-sen University. H1 ESCs were cultured in mTeSR1 medium (Stem Cell Technologies), and passaged by mechanical dissociation every 4-6 days. The two GS iPSC lines (GS-iPSC1 and GS-iPSC3) were cultured as previously described 29. GS HLCs were maintained in HCM (Lonza) supplemented with 2% Fetal Bovine Serum (FBS; Thermo Fisher Scientific). All cells were incubated at 37 °C in a humidified atmosphere with 5% CO_2_.

### Hepatocyte differentiation* in vitro*

About 1×10^5^ cells/cm^2^ of GS-iPSCs or 5×10^5^ cells/cm^2^ of human ESCs were harvested with Accutase (Thermo Fisher Scientific) and plated onto Matrigel-coated culture dishes. DE differentiation was initiated the next day using RPMI 1640 (Thermo Fisher Scientific) together with 2% B-27 Supplement (Thermo Fisher Scientific, see Table S2), 3 μM CHIR99021 (Selleck) and 100 ng/mL activin A (Peprotech). Following 24-h treatment, CHIR99021 and activin A were removed and the cells were incubated in RPMI 1640/B-27 basal medium alone for another 24 h. Subsequently, differentiated cells were cultured in RPMI 1640/B-27 basal medium supplemented with 100 ng/ml activin A for 2 days. To induce hepatocyte differentiation, GS DE cells were detached using Accutase and seeded at a density of 1×10^4^ cells/ cm^2^ on Matrigel-coated dishes in the presence of differentiation medium (advanced F12, 8% FBS, 1% GlutaMAX supplement, and 1% Penicillin-Streptomycin; Thermo Fisher Scientific), supplemented with 100 ng/mL HGF (Peprotech) for 7 days. For human cultures, DE cells were seeded at a density of 1×10^5^ cells / cm^2^ in differentiation medium (advanced F12, 8% KSR, 1% GlutaMAX supplement, and 1% Penicillin-Streptomycin) (Thermo Fisher Scientific) containing 100 ng/ml of HGF (Peprotech) and 10 µM Rock Inhibitor Y-27632 (MCE). After one day, the cells were cultured for another 6 days in differentiation medium containing 100 ng/mL of HGF, with daily medium change. In the final stage, cells were further matured in HCM (Lonza) containing 20 ng/mL oncostatin M (Peprotech), 100 nM of dexamethasone (Sigma) and 2% FBS for 5 days. The differentiated HLCs were subsequently maintained in HCM containing 2% FBS, with the medium being changed twice per week.

### Cold exposure and hypoxia experiments

Cold exposure experiments were carried out in the same way as previously described 29. Briefly, HLCs cultured under normal conditions were used as 37 °C controls. In the cold treatment groups, normal culture medium was replaced with Hibernate-A medium (Thermo Fisher Scientific) supplemented with the same doses of growth factors and other supplements. Cells in the hibernate-medium were incubated at room temperature for 10 min before being transported to a 4 °C refrigerator for specified durations. Cells in the rewarmed groups were taken out of the refrigerator and allowed to rewarm to room temperature for 10 min. Then hibernate-medium was replaced with normal culture medium, and the cells were maintained normally for the designated time. For hypoxia experiments, HLCs cultured at normal condition were used as normal controls. In hypoxia treatment groups, normal culture medium was replaced with low-glucose Dulbecco's Modified Eagle Medium (Thermo Fisher Scientific) supplemented with the same amounts of growth factors and other supplements. Cells in this group were incubated at room temperature for 10 min before being exposed to a hypoxic environment with 5% CO_2_, 1% O_2_, and 94% N_2_ in a 37 °C incubator for the specified duration. The hypoxic medium of the cultures in the re-oxygenation group was replaced with the normal culture medium, and then the cells were cultured at normal conditions for the designated time.

### Sample preparation and extraction for metabolomics

For GS HLCs, cells were subjected to cold exposure and rewarming, or hypoxia and re-oxygenation as described in the above section. Then, the incubation mediums were quickly discarded, 1 mL of pre-cooled extractant (80% methanol aqueous solution) was added and the cells were harvested. The mixture was vortexed for 2 min, frozen in liquid nitrogen for 3 min, and allowed to thaw for 5 min on ice. This step was repeated for 3 times, then the mixture was centrifuged at 12000 rpm, 4 °C for 10 min. Finally, 200 μL of supernatant was transferred for on-board analysis. For human liver samples, they were thawed on ice. About 50 ±2 mg tissue per sample was mixed with cold steel balls and homogenized at 30 Hz for 3 min. Then 1 mL 70% methanol with internal standard extract was added to the homogenized samples. Samples were vortexed for 5 min, and then centrifuge at 12,000 rpm, 4 °C for 10 min. After centrifugation, 400 μL of supernatant was transferred to the corresponding EP tube and stored at -20 °C overnight. Following centrifuging at 12000 rpm at 4 °C for 3 min, 200 μL of supernatant was transferred for on-board analysis.

### Metabolomic analyses

For T3 ultra performance liquid chromatography (UPLC), the sample extracts were analyzed using an LC-ESI-MS/MS system (ExionLC AD System, Sciex; QTRAP® System, Sciex).The analytical conditions were as follows, UPLC: column, Waters ACQUITY UPLC HSS T3 C18 (1.8 µm, 2.1 mm*100 mm); column temperature, 40 °C; flow rate, 0.4 mL/min; injection volume, 2μL; solvent system, water (0.1% formic acid): acetonitrile (0.1% formic acid); gradient program, 95:5 V/V at 0 min, 10:90 V/V at 11.0 min, 10:90 V/V at 12.0 min, 95:5 V/V at 12.1 min, 95:5 V/V at 14.0 min. The analytical conditions for Amide UPLC were as follows, UPLC: column, Waters ACQUITY UPLC BEH Amide 1.7 µm, 2.1 mm*100 mm; column temperature, 40 °C; flow rate, 0.4 mL/min; injection volume, 2 μL; solvent system, water (25 mM Ammonium formate/ 0.4% Ammonia): acetonitrile; gradient program, 10:90 V/V at 0 min, 40:60 V/V at 9.0 min, 60:40 V/V at 10.0 min, 60:40 V/V at 11.0 min, 10:90 V/V at 11.1 min, 10:90 V/V at 15.0 min. T3 and Amide have the same mass spectrometry parameters. LIT and triple quadrupole (QQQ) scans were acquired on a triple QTRAP® LC-MS/MS System equipped with an ESI Turbo Ion-Spray interface, operating in positive and negative ion mode and controlled by Analyst 1.6.3 software (Sciex). The ESI source operation parameters were as follows: source temperature 500 °C; ion spray voltage (IS) 5500 V (positive), -4500 V (negative); ion source gas I (GSI), gas II (GSII), and curtain gas (CUR) were set at 55, 60, and 25.0 psi, respectively; the collision gas (CAD) was high. Instrument tuning and mass calibration were performed with 10 and 100 μmol/L polypropylene glycol solutions in QQQ and LIT modes, respectively. A specific set of MRM transitions were monitored for each period according to the metabolites eluted within this period.

For analysis, significantly altered metabolites between groups were determined by VIP ≥ 1 and absolute Log_2_FC (fold change) ≥ 1. VIP values were extracted from orthogonal partial least squares discriminant analysis (OPLS-DA) result, which was generated using R package MetaboAnalystR (version 5.0). Data was log transformed (log2) and mean centering before OPLS-DA. To avoid overfitting, a permutation test (200 permutations) was performed. Identified metabolites were annotated using KEGG Compound database, then annotated metabolites were mapped to KEGG Pathway database. Significantly enriched pathways are identified with a hypergeometric test's *p*-value for a given list of metabolites. Unsupervised principal component analysis (PCA) was performed by statistics function prcomp within R language (www.r-project.org). The data was unit variance scaled before unsupervised PCA. Volcano plots, heatmaps and violin plots were created using Hiplot (https://hiplot.com.cn), a comprehensive web platform for scientific data visualization. For volcano plots, the horizontal line was at -log10 (*p* value) = 0.25 and the vertical line at |Log_2_FC| = 1.2. For heatmap showing significantly altered metabolites among groups in GS HLCs, an absolute Log_2_FC (fold change) ≥ 1.2 was selected. For heatmap showing significantly regulated metabolites between groups of human donor livers, an absolute Log_2_FC (fold change) ≥ 3 was selected. The Pearson correlation coefficients (PCC) between samples were calculated by the Cor function in R. Metabolites with a *p* value < 0.05 and | PCC | > 0.8 were selected for chord plots to depict associations between differentially expressed metabolites.

### RNA isolation and Real-time (RT) qPCR analysis

Total RNA was extracted from the cells with RNeasy Mini kit (Qiagen, Hilden, Germany) according to the manufacturer's instruction. The total RNA concentration was quantified by Nanodrop Spectrophotometer 2000 (Thermo Fisher Scientific). RNA from each sample was reverse transcribed with high-capacity cDNA reverse transcription kit (Roche, Germany). RT qPCR was performed using the SYBR qPCR Master Mix (Vazyme, China) in the 7900 HT Fast RT qPCR system (Roche, Germany). Relative folding changes of gene expression were identified with the 2^-ΔΔCt^ method. Primers used for RT qPCR are shown in Table S3.

### Transfection of siRNA

GS *ALAS1* siRNAs used are listed in Table S3. GS *ALAS1* siRNAs were transfected into GS HLCs with Lipofectamine 3000 (Thermo Fisher Scientific) following manufacturer's instruction. After 12 h at 37 °C the transfection medium was replaced with fresh culture medium. GS HLCs were further cultured for 24 h before cold exposure. Cold exposure and rewarming experiments were performed as described above.

### Immunofluorescence staining and imaging

Cells were fixed with 4% paraformaldehyde (Sigma) for 15 min at room temperature. After 0.2% Triton X-100 treatment, cells were incubated with primary antibodies diluted in 3% BSA/ 0.1% Triton X-100 overnight at 4 °C, followed by incubation with Alexa Fluor-conjugated secondary antibodies (Invitrogen) for 1 h at room temperature. Cells were mounted using a mounting medium with 4,6-diamidino-2-phenylindole (DAPI) (Sigma). Images were acquired with LSM880 confocal microscope (Carl Zeiss). The primary antibodies are listed in Table S4.

### Western blotting

Cells were lysed in Mammalian Protein Extraction Reagent (Thermo Fisher Scientific) supplemented with protease inhibitor cocktail (Thermo Fisher Scientific) for 10 min on ice. Cell lysates were centrifuged at 12,000 g for 10 min at 4 °C. The protein concentration in the supernatants was determined by the Pierce BCA Protein Assay Kit (Thermo Fisher Scientific) according to the manufacturer's protocol. Protein samples were resolved by SDS-PAGE and transferred to polyvinylidene fluoride membranes (Millipore, Germany). The membranes were blocked with 5% skimmed milk in TBST (50 mM Tris, 0.5 M NaCl, 0.05% Tween-20, pH 7.4) for 1 h and incubated with primary antibodies at 4 °C overnight, followed by incubation with horseradish peroxidase (HRP)-conjugated secondary antibodies for 1 h at room temperature the next day. The bands were visualized using the Immobilon Western Chemiluminescent HRP Substrate (Millipore, Germany). Images were analyzed using Image-Pro plus software. The primary antibodies are listed in Table S4.

### Dot blotting

Supernatant of cell cultures at different stages was concentrated by Amicon Ultra 15 mL centrifugal devices (Millipore, Germany). A volume of 5 µL of each sample were spotted onto the polyvinylidene fluoride membranes, then the membranes were dried for 1 h at room temperature. The membranes were blocked with 5% dry milk in TBST (50 mM Tris, 0.5 M NaCl, 0.05% Tween-20, pH 7.4) for 1 h at room temperature, and incubated with anti-ALB primary antibody (Table S4) for 1 h at room temperature in 0.1% BSA in TBST, followed by incubation with HRP-secondary antibodies for 1 h at room temperature. Blots were detected with Immobilon Western Chemiluminescent HRP Substrate (Millipore, Germany). Images were analyzed using Image-Pro plus software.

### Periodic acid-Schiff staining (PAS), cellular uptake and release of Indocyanine Green (ICG) and BODIPY staining

For glycogen detection, cells were fixed with 4% paraformaldehyde for 10 min, and stained using a PAS staining kit (Sigma) according to the manufacturer's instructions. ICG staining has been shown to be a valuable test of differentiated HLCs from stem cells. Cellular uptake of ICG (Sigma) was performed by incubating cells in the culture medium with 1 mg/mL ICG for 1 h at 37 ˚C. ICG was cleared after an overnight incubation. Images were taken by ECLIPSE E100 (Nikon, Tokyo, Japan) using phase contrast lenses. To detect lipid droplets, HLCs were fixed with 4% paraformaldehyde and permeabilized with 0.2% Triton X-100 in PBS. Cells were then incubated with BODIPY 493/503 (Thermo Fisher Scientific) for 30 min in the dark at room temperature, and mounted using mounting medium containing DAPI (Sigma).

### Cell viability analysis

Cell Counting Kit-8 (Dojindo Molecular Technologies) was used to determine the number of viable cells according to the manufacturer's protocol. A cell suspension containing 1×10^4^ cells/100 µL medium was added to each well in a 96-well plate (Thermo Fisher Scientific). Cells were incubated at 4 ℃ for 24 h, then at 37 ℃ for 2 h. 5-ALA (0.2-5 mM) was added during cold exposure. The fold changes were calculated using baseline values of control cells as a reference (set to 1).

### Flow cytometry analysis

Cells were dissociated with Accutase (Thermo Fisher Scientific). The cells were fixed with 4% paraformaldehyde (Sigma) for 15 min and permeabilized with 0.5 % Triton X-100 for 10 min at room temperature. The cells were subsequently incubated with primary antibodies for 1 h at 4 °C, followed by a 30 min' incubation with the fluorochrome-labeled secondary antibodies at room temperature. For detection of cell death, cells were stained with 2 ug/mL propidium iodide (PI,Sigma) for 15 min in the dark at room temperature. Then the cells were analyzed using CytoFLEX LX and Cytoexpert software (Beckman, USA). Primary antibodies used in flow cytometry are in Table S4.

### Live cell imaging and analysis of mitochondrial membrane potential (Δψm)

The change of Δψm was measured using JC-1 (Beyotime Biotechnology, China) by quantifying the fluorescence emission shift from green (529 nm) monomers to red (590 nm) aggregates. Prior to analysis, cells were incubated for 20 min at 37 °C to ensure the formation of an equilibrium between JC-1 aggregates and monomers. The medium containing JC-1 was removed, and fresh pre-warmed hibernate-medium was added. After 4-h of cold preservation, hibernate-medium was changed to normal medium, and the cells were imaged under LSM880 system (Zeiss). During the Z-stack time-lapse confocal imaging, cells in the field of view were refocused. The same Z-stack, same position and time-lapse confocal images were taken for 2 h. A maximum intensity projection of the Z-stack images was produced for data analysis. At least 5 different well-focused images per experiment were measured. The data are expressed as a fold increase in the red/green ratio.

### Live cell imaging and analysis of mitochondrial ROS

MitoNeoD (MedKoo Biosciences, Inc, USA) is a novel mitochondrion-targeted probe for superoxide production. Cells were incubated in hibernate-medium containing 2.5 μM MitoNeoD for 20 min in the dark at room temperature. Following 4-h cold exposure, the hibernate-medium was changed to normal medium and the cells were imaged and analyzed as described above. For high content imaging, 96-well plates were read in a bioluminescence plate reader (Operetta CLS, PerkinElmer). At least 5 different well-focused images of cells per experiment were measured.

### NAD^+^/NADH, NADP^+^/NADPH and ATP assays

The NAD/NADH-Glo Assay kit (Promega) and the NADP/NADPH-Glo Assay kit (Promega) were used according to the manufacturer's instructions. Briefly, 1×10^5^ cells were extracted in 100 μL of ice-cold lysis buffer (1% DTAB in 0.2 N of NaOH diluted 1:1 with PBS). To measure NAD^+^ or NADP^+^, 50 μL of lysates were transferred to PCR tubes supplemented with 25 uL of 0.4 N HCl; another 50 μL of lysates were transferred to PCR tubes for NADH or NADPH measurement. All samples were incubated at 60 °C for 15 min. After the plate was cooled to room temperature for 10 min, 25 μL of 0.5 M Trizma base solution was added to the wells for NAD^+^ or NADP^+^ measurements, and 50 μL of a 1:1 mixture of 0.4 N HCl/ 0.5 M Trimza base solution was added to the wells for NADH or NADPH measurements, totaling 100 μL for all samples. After gently mixing 100 μL of NAD^+^/NADH-Glo or NADP^+^/NADPH-Glo detection reagent into each well and incubating for 30 min at room temperature, the luminescence levels were measured using a plate reader. ATP was detected using a luciferase assay kit (Promega) with a 590 nm luminescence measurement. The data are expressed as relative fluorescence levels adjusted for protein levels, with the mean values measured at 37 °C set to 1.

### Porphobilinogen (PBG) Assay

Cells were homogenized in lysis buffer (0.6 M Tris, 0.1M EDTA, pH 8.2). Then 100 μL sample was mixed with 100 μL fresh Modified Ehrlich reagent (dissolve 1.0 g p-dimethylamino benzaldehyde (Sigma) in 30 mL glacial acetic acid (Sigma) and mix with 8 mL 70% perchloric acid (Sigma), then bring to 50 mL with glacial acetic acid) and incubated for 10 min at 37 ℃. Concentration of PBG was determined spectrophotometrically at 37 °C in absorbance at 555 nm.

### DHODH activity and Complex III activity

DHODH activity and complex III activity were measured by spectrophotometry as previously described 59. DHODH activity was determined spectrophotometrically at 37 °C by monitoring the decrease in absorbance at 600 nm of reduced 2,6-dichlorophenol-indophenol (DCPIP). Briefly, the reaction was initiated with 20 mM dihydroorotate (Sigma) in 1 ml of standard reaction buffer supplemented with 50 μM DCPIP (Sigma), 2 μg of rotenone (Sigma), 2 μg of antimycin A (Sigma), 5 mM NaN_3_ (Sigma) and 0.1 mg of whole-cell lysate. The reaction was stopped by the addition of 2 μg leflunomide. Complex III activity was evaluated spectrophotometrically at 37 °C by monitoring the increase in absorbance at 550 nm of cytochrome c. Briefly, the reaction was initiated with 5 mM decylubiquinone (Sigma) to 1 mL of standard reaction buffer supplemented with 2 μg of rotenone (Sigma), 5 mM NaN_3_, 60 μM cytochrome c (Sigma) and 0.1 mg of whole-cell lysate. The reaction was stopped by the addition of 2 μg antimycin A (Sigma). One unit was defined as nmol· min ^-1^· μg ^-1^ of protein. The fold changes were calculated using baseline values of control cells as a reference (set to 1).

### Complex IV activity

Complex IV activity was measured by the Complex IV activity assay kit (Acmec, China) according to the manufacturer's instructions. Briefly, cells were homogenized in an extraction buffer. The homogenate was centrifuged at 600 g for 10 min, and then the supernatant was further centrifuged at 11,000 g for 10 min. The pellet was suspended in an extraction buffer and used as a mitochondrial fraction. Protein concentrations were determined by the Pierce BCA Protein Assay Kit (Thermo Fisher Scientific). Complex IV was measured by the decrease in absorption at 550 nm. One unit of complex IV activity was defined as the oxidization of 1.0 nmol of reduced cytochrome c per min in 1 mg of protein. The fold changes were calculated using the baseline values of control cells as a reference (set to 1).

### Isolated perfused rat liver (IPRL)

Male Sprague-Dawley Rats were purchased from Beijing SiPeiFu biotechnology co., LTD. Rats had free access to water and standard chow before surgery. All experiments were approved by the Institutional Animal Care and Use Committee at the Third Affiliated Hospital of Sun Yat-Sen University. After overnight fasting, rats (260-290 g) were anesthetized by inhalation of isoflurane and heparinized with 50 IU heparin. Bile duct was cannulated with a PE-10 catheter (Yuyan, China) and portal vein was cannulated with 22-G Introcan catheter (Braun, Germany). Then the liver was flushed with normal saline and UW solution or UW solution supplemented with 2 mM 5-aminolevulinic acid (ALA) hydrochloride (Macklin, China), then harvested (n = 5-6 per group). Livers were stored in static preservation solution at 4 ℃ for 48 h. The IPRL system (Hugo Sachs Elektronik, Germany) was set up in a circulatory manner. A circulating volume of 250 mL Krebs-Henseleit bicarbonate buffer (Sigma) was prepared and oxygenated by a fiber oxygenator with mixed gas of 95% O_2_ and 5% CO_2_, reaching a partial oxygen pressure over 500 mmHg. Livers were equilibrated at room temperature for 30 min after cold storage, and perfused at 37 ℃. Flow rate was set at pressure-controlled mode with a constant portal venous pressure (PVP) of 12 mmHg. Flow rate and portal venous pressure were monitored and recorded automatically. Portal venous resistance (PVR) was calculated as follows 60: PVR (mmHg/min × min × g liver) = PVP (12 mmHg)/ portal flowrate (mL × min × g liver). Bile was collected and expressed as μL/g liver after 2-h perfusion. Perfusate was collected every 20 min until the end of perfusion. Levels of aspartate transaminase (AST), alanine transaminase (ALT) and lactate dehydrogenase (LDH) in perfusate were measured at the Department of Laboratory Medicine in the Third Affiliated Hospital of Sun Yat-Sen University using HITACHI 7600 Series (Japan). Liver samples were snapped frozen or fixed in 4% paraformaldehyde.

### Histology

Fixed samples were embedded in paraffin or dehydrated in 15% and 30% sucrose in PBS. Paraffin sections (3 μm) were stained with hematoxylin-eosin and photographed under light microscope. Dehydrated tissue blocks were submerged in OCT and frozen. Frozen sections (8 μm) were stained with *In situ* Cell Death Detection Kit (Roche) according to the manufacturer's protocol. Briefly, frozen slides were brought to room temperature for 30 min. Then slides were washed in PBS and permeabilized before incubation on terminal-deoxynucleotidyl transferase mediated nick end labeling (TUNEL) reaction mixture for 60 min at 37 ℃. Then slides were stained with DAPI, mounted, and inspected by the LSM880 confocal microscope. At least 5 random fields (40x) per sample were captured to calculate the percentage of TUNEL-positive nuclei.

### RNA-Seq

Total RNA extracts were prepared from isolated perfused rat livers in UW solution group or UW + 5-ALA group. RNA-Seq was performed on an Illumina Novaseq platform according to the manufacturer's instructions. More details are described in supplementary methods.

### Statistical analysis

All data were obtained from at least three independent experiments, presented as mean ± SEM. Statistical analysis was performed by Student's *t*-test or ANOVA using GraphPad (GraphPad Prism Software Inc, San Diego, CA, USA). *P* < 0.05 was considered statistically significant.

## Supplementary Material

Supplementary figures and tables, methods.

Supplementary data.

## Abbreviations

AAT: α1-antitrypsin
AFP: alpha fetoprotein
5-ALA: 5-Aminolevulinate
ALAS1: 5-aminolevulinate synthase 1
ALB: albumin
ALT: alanine transaminase
APOA1: apolipoprotein A1
AST: aspartate transaminase
CK18: cytokeratin 18
CK19: cytokeratin 19
CYP: cytochrome P450
DAPI: 4,6-diamidino-2-phenylindole
DE: definitive endoderm
DHODH: dihydroorotate dehydrogenase
ESC: embryonic stem cell
FOXA2: forkhead box a2
GATA4: GATA binding protein 4
GS: thirteen-lined ground squirrel
HLC: hepatocyte-like cell
4-HNE: 4-hydroxynonenal
HNF4A: hepatocyte nuclear factor 4α
ICG: indocyanine green
iPSC: induced pluripotent stem cell
IR: ischemia/reperfusion
LDH: lactate dehydrogenase
NANOG: nanog homeobox
NRF2: nuclear factor erythroid 2-related factor 2
OCT4: POU class 5 homeobox 1
PAS: Periodic Acid-Schiff
PBG: porphobilinogen
PI: propidium iodide
RET: reverse electron transport
ROS: reactive oxygen species
SOX17: sex-determining region y-box 17
TBX3: t-box transcription factor 3
TTR: transthyretin
UW: University of Wisconsin
