# Supplementary material for: 5-Aminolevulinate improves metabolic recovery and cell survival of the liver following cold preservation
Source: Theranostics. 2022 Mar 21;12(6):2908–27. doi: 10.7150/thno.69446 (PMC8965473; doi:10.7150/thno.69446)
Supplement: Supplementary file 1 — Supplementary figures and tables, methods. [file thnov12p2908s1.pdf]

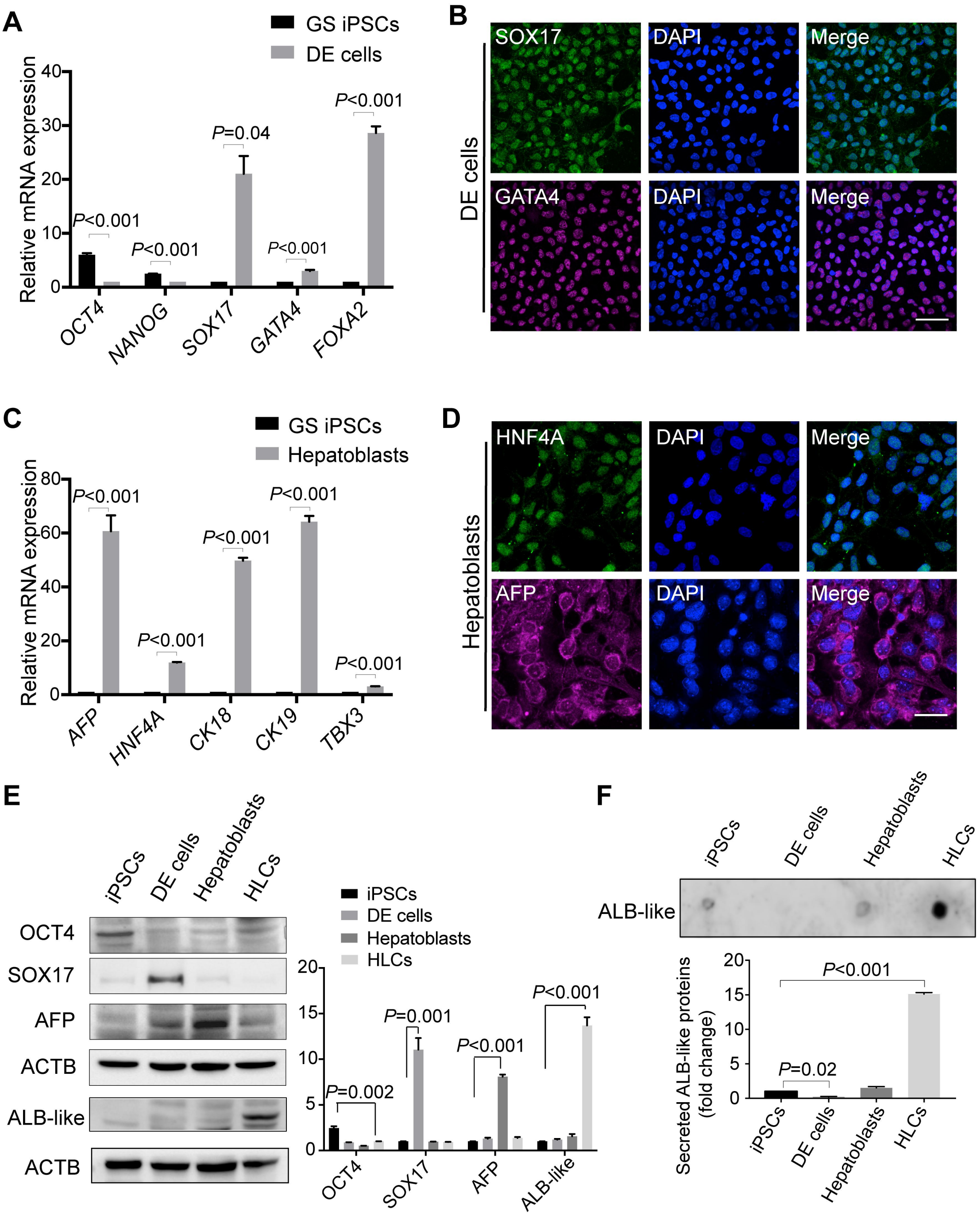
A
GS iPSCs
DE cells
Relative mRNA expression
P<0.001
P<0.001
P=0.04
P<0.001
P<0.001
OCT4
NANOG
SOX17
GATA4
FOXA2
B
DE cells
SOX17
DAPI
Merge
GATA4
DAPI
Merge
C
GS iPSCs
Hepatoblasts
Relative mRNA expression
P<0.001
P<0.001
P<0.001
P<0.001
P<0.001
AFP
HNF4A
CK18
CK19
TBX3
D
Hepatoblasts
HNF4A
DAPI
Merge
AFP
DAPI
Merge
E
iPSCs
DE cells
Hepatoblasts
HLCs
OCT4
SOX17
AFP
ACTB
ALB-like
ACTB
iPSCs
DE cells
Hepatoblasts
HLCs
P=0.002
P=0.001
P<0.001
P<0.001
OCT4
SOX17
AFP
ALB-like
F
iPSCs
DE cells
Hepatoblasts
HLCs
ALB-like
Secreted ALB-like proteins (fold change)
P<0.001
P=0.02
iPSCs
DE cells
Hepatoblasts
HLCs

**Figure S1. Expression of key markers at each differentiation stage of GS iPSCs into HLCs.** (**A**) Gene expression of pluripotency (*OCT4* and *NANOG*) and definitive endoderm (DE) markers (*SOX17*, *GATA4* and *FOXA2*) in DE cells (n = 3). GS iPSCs were used as control, and the expression is normalized to that of *ACTB*. (**B**) Immunofluorescence staining of SOX17 and GATA4 in DE cells at the endpoint of stage I (n = 5); scale bar: 100 µm. (**C**) RT qPCR analysis of hepatoblast markers (*AFP*, *HNF4A*, *CK18*, *CK19* and *TBX3*) at the stage II endpoint (n = 3). GS iPSCs were used as control, and the expression is normalized to that of *ACTB*. (**D**) Immunofluorescence staining of AFP and HNF4A in hepatoblasts at the endpoint of stage II (n = 5); scale bar: 50 µm. (**E**) Representative immunoblots and quantification of OCT4, SOX17, AFP and ALB-like proteins in different stages of GS HLC differentiation. ACTB was used as the loading control (n = 3). (**F**) Representative dot blot of secreted ALB-like proteins in the culture supernatant during differentiation (n = 3). Data are expressed as mean ± SEM; Student's *t*-test comparing to GS iPSCs (A, C and E); one-way ANOVA followed by Dunnett multiple-comparisons test versus GS iPSCs (F); *P* values are indicated.

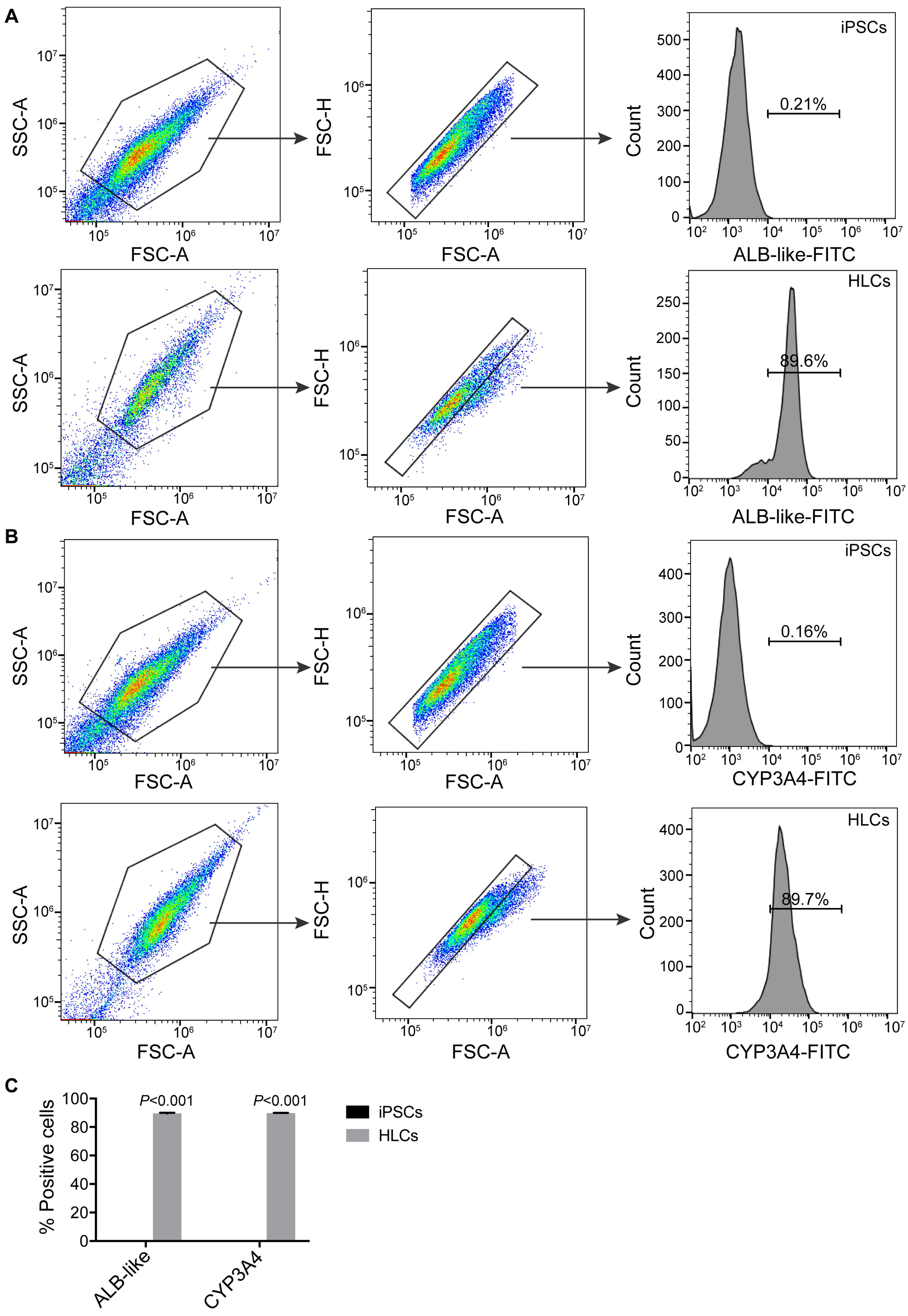
A
B
C
SSC-A
FSC-A
FSC-H
Count
ALB-like-FITC
CYP3A4-FITC
iPSCs
HLCs
0.21%
89.6%
0.16%
89.7%
% Positive cells
P<0.001
P<0.001
ALB-like
CYP3A4

**Figure S2. Gating strategy of flow cytometry in characterizing GS iPSCs and HLCs.** Gating strategy of ALB-like (**A**) and CYP3A4 (**B**) in GS iPSCs and GS HLCs analyzed by flow cytometry. (**C**) Percentage of ALB-like- and CYP3A4-positive cells in GS iPSCs and HLCs (n = 3). Data are expressed as mean ± SEM; Student's *t*-test between two comparisons; *P* values are indicated.

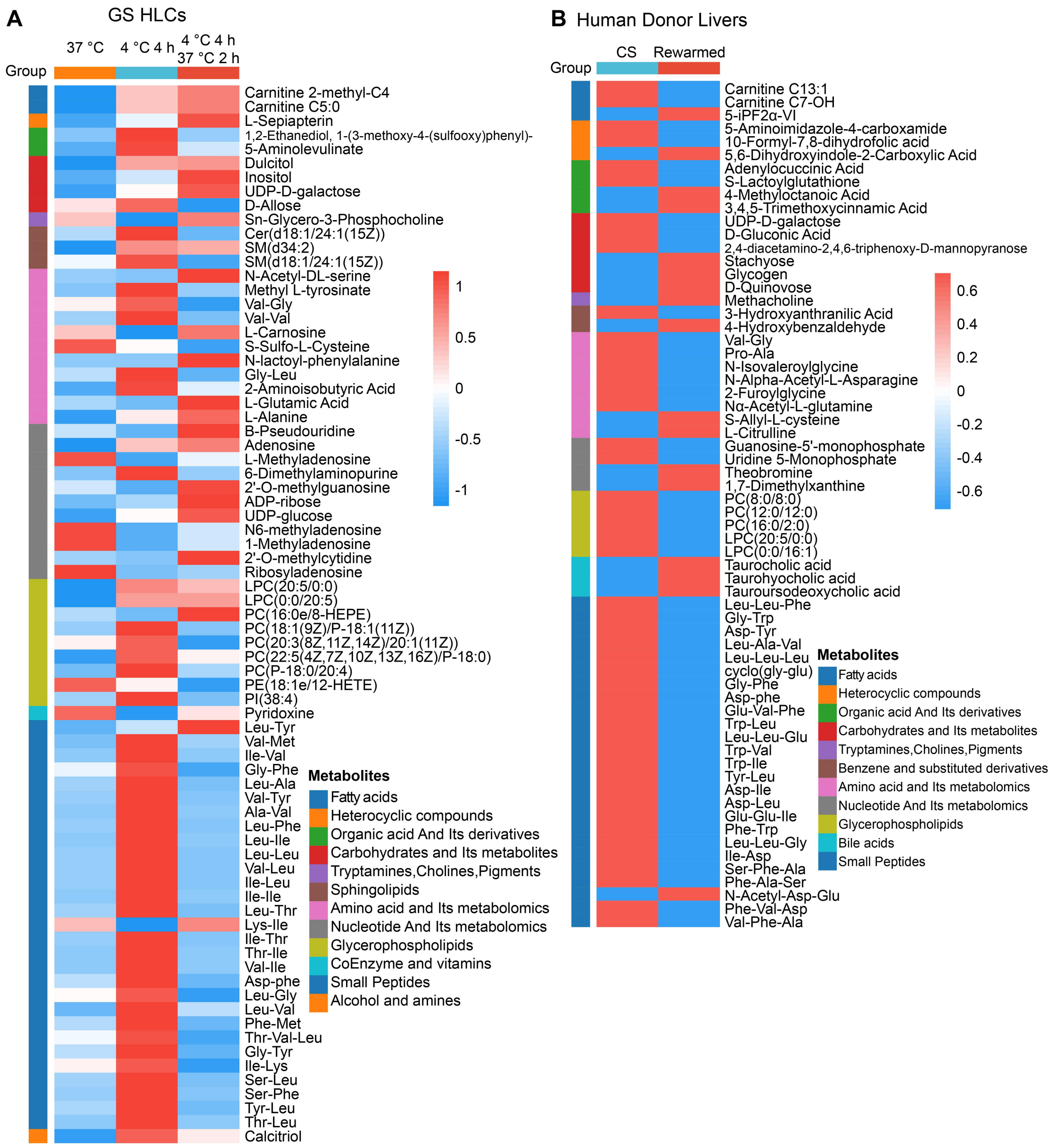
A
GS HLCs
37 °C
4 °C 4 h
4 °C 4 h 37 °C 2 h
Group
Carnitine 2-methyl-C4
Carnitine C5:0
L-Sepiapterin
1,2-Ethanediol, 1-(3-methoxy-4-(sulfooxy)phenyl)-
5-Aminolevulinate
Dulcitol
Inositol
UDP-D-galactose
D-Allose
Sn-Glycero-3-Phosphocholine
Cer(d18:1/24:1(15Z))
SM(d34:2)
SM(d18:1/24:1(15Z))
N-Acetyl-DL-serine
Methyl L-tyrosinate
Val-Gly
Val-Val
L-Carnosine
S-Sulfo-L-Cysteine
N-lactoyl-phenylalanine
Gly-Leu
2-Aminoisobutyric Acid
L-Glutamic Acid
L-Alanine
B-Pseudouridine
Adenosine
L-Methyladenosine
6-Dimethylaminopurine
2'-O-methylguanosine
ADP-ribose
UDP-glucose
N6-methyladenosine
1-Methyladenosine
2'-O-methylcytidine
Ribosyladenosine
LPC(20:5/0:0)
LPC(0:0/20:5)
PC(16:0e/8-HEPE)
PC(18:1(9Z)/P-18:1(11Z))
PC(20:3(8Z,11Z,14Z)/20:1(11Z))
PC(22:5(4Z,7Z,10Z,13Z,16Z)/P-18:0)
PC(P-18:0/20:4)
PE(18:1e/12-HETE)
PI(38:4)
Pyridoxine
Leu-Tyr
Val-Met
Ile-Val
Gly-Phe
Leu-Ala
Val-Tyr
Ala-Val
Leu-Phe
Leu-Ile
Leu-Leu
Val-Leu
Ile-Leu
Ile-Ile
Leu-Thr
Lys-Ile
Ile-Thr
Thr-Ile
Val-Ile
Asp-phe
Leu-Gly
Leu-Val
Phe-Met
Thr-Val-Leu
Gly-Tyr
Ile-Lys
Ser-Leu
Ser-Phe
Tyr-Leu
Thr-Leu
Calcitriol
1
0.5
0
-0.5
-1
Metabolites
Fatty acids
Heterocyclic compounds
Organic acid And Its derivatives
Carbohydrates and Its metabolites
Tryptamines,Cholines,Pigments
Sphingolipids
Amino acid and Its metabolomics
Nucleotide And Its metabolomics
Glycerophospholipids
CoEnzyme and vitamins
Small Peptides
Alcohol and amines
B Human Donor Livers
CS
Rewarmed
Group
Carnitine C13:1
Carnitine C7-OH
5-iPF2α-VI
5-Aminoimidazole-4-carboxamide
10-Formyl-7,8-dihydrofolic acid
5,6-Dihydroxyindole-2-Carboxylic Acid
Adenylocuccinic Acid
S-Lactoylglutathione
4-Methyloctanoic Acid
3,4,5-Trimethoxycinnamic Acid
UDP-D-galactose
D-Gluconic Acid
2,4-diacetamino-2,4,6-triphenoxy-D-mannopyranose
Stachyose
Glycogen
D-Quinovose
Methacholine
3-Hydroxyanthranilic Acid
4-Hydroxybenzaldehyde
Val-Gly
Pro-Ala
N-Isovaleroylglycine
N-Alpha-Acetyl-L-Asparagine
2-Furoylglycine
Nα-Acetyl-L-glutamine
S-Allyl-L-cysteine
L-Citrulline
Guanosine-5'-monophosphate
Uridine 5-Monophosphate
Theobromine
1,7-Dimethylxanthine
PC(8:0/8:0)
PC(12:0/12:0)
PC(16:0/2:0)
LPC(20:5/0:0)
LPC(0:0/16:1)
Taurocholic acid
Taurohyocholic acid
Tauroursodeoxycholic acid
Leu-Leu-Phe
Gly-Trp
Asp-Tyr
Leu-Ala-Val
Leu-Leu-Leu
cyclo(gly-glu)
Gly-Phe
Asp-phe
Glu-Val-Phe
Trp-Leu
Leu-Leu-Glu
Trp-Val
Trp-Ile
Tyr-Leu
Asp-Ile
Asp-Leu
Glu-Glu-Ile
Phe-Trp
Leu-Leu-Gly
Ile-Asp
Ser-Phe-Ala
Phe-Ala-Ser
N-Acetyl-Asp-Glu
Phe-Val-Asp
Val-Phe-Ala
0.6
0.4
0.2
0
-0.2
-0.4
-0.6
Metabolites
Fatty acids
Heterocyclic compounds
Organic acid And Its derivatives
Carbohydrates and Its metabolites
Tryptamines,Cholines,Pigments
Benzene and substituted derivatives
Amino acid and Its metabolomics
Nucleotide And Its metabolomics
Glycerophospholipids
Bile acids
Small Peptides

**Figure S3. Metabolomic profiles of GS HLCs and human donor livers.** (**A**) Heatmap showing differential metabolites of GS HLCs from the 37 °C, 4 °C 4 h and 4 °C 4 h-37 °C 2 h groups (n = 3 per group). (**B**) Heatmap showing differential metabolites of human donor livers during cold storage (CS) versus post-transplantation (Rewarmed; n = 10 per group).

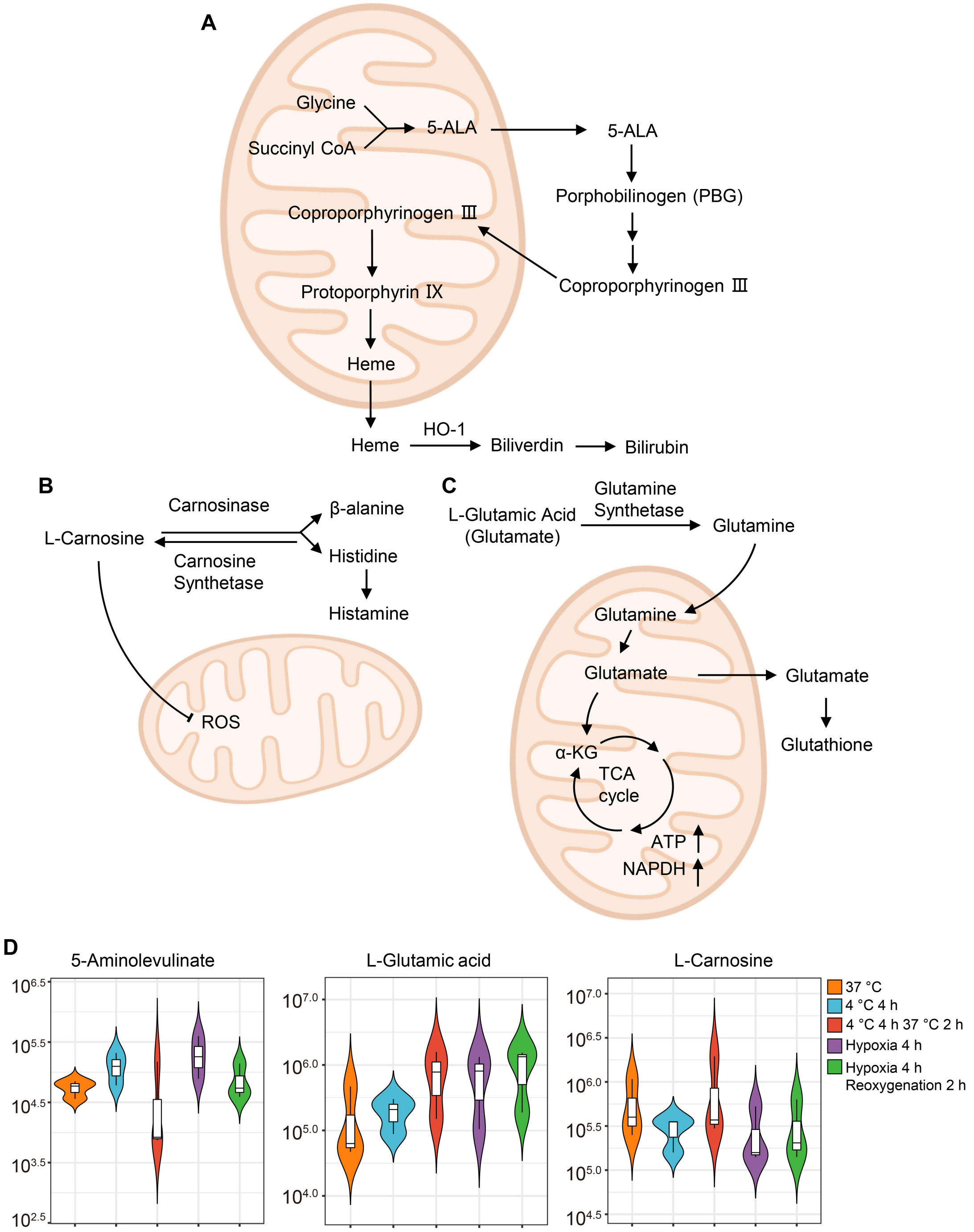
A
Glycine
Succinyl CoA
5-ALA
5-ALA
Porphobilinogen (PBG)
Coproporphyrinogen Ⅲ
Coproporphyrinogen Ⅲ
Protoporphyrin Ⅸ
Heme
Heme
HO-1
Biliverdin
Bilirubin
B
L-Carnosine
Carnosinase
β-alanine
Histidine
Carnosine Synthetase
Histamine
ROS
C
L-Glutamic Acid (Glutamate)
Glutamine Synthetase
Glutamine
Glutamine
Glutamate
Glutamate
Glutathione
α-KG
TCA cycle
ATP
NAPDH
D
5-Aminolevulinate
L-Glutamic acid
L-Carnosine
$10^{6.5}$
$10^{5.5}$
$10^{4.5}$
$10^{3.5}$
$10^{2.5}$
$10^{7.0}$
$10^{6.0}$
$10^{5.0}$
$10^{4.0}$
$10^{7.0}$
$10^{6.5}$
$10^{6.0}$
$10^{5.5}$
$10^{5.0}$
$10^{4.5}$
37 °C
4 °C 4 h
4 °C 4 h 37 °C 2 h
Hypoxia 4 h
Hypoxia 4 h Reoxygenation 2 h

**Figure S4**. **The Metabolic pathways and temperature/oxygen supply-induced changes of selected metabolites.** (**A-C**) Schematic diagrams showing pathways of selected metabolites involved in mitochondrial metabolism (A, 5-aminolevulinate; B, L-Carnosine; C, L-Glutamic acid). (**D**) Violin Plots showing abundance of 5-aminolevulinate, L-carnosine and L-glutamic acid in GS HLCs at annotated conditions.

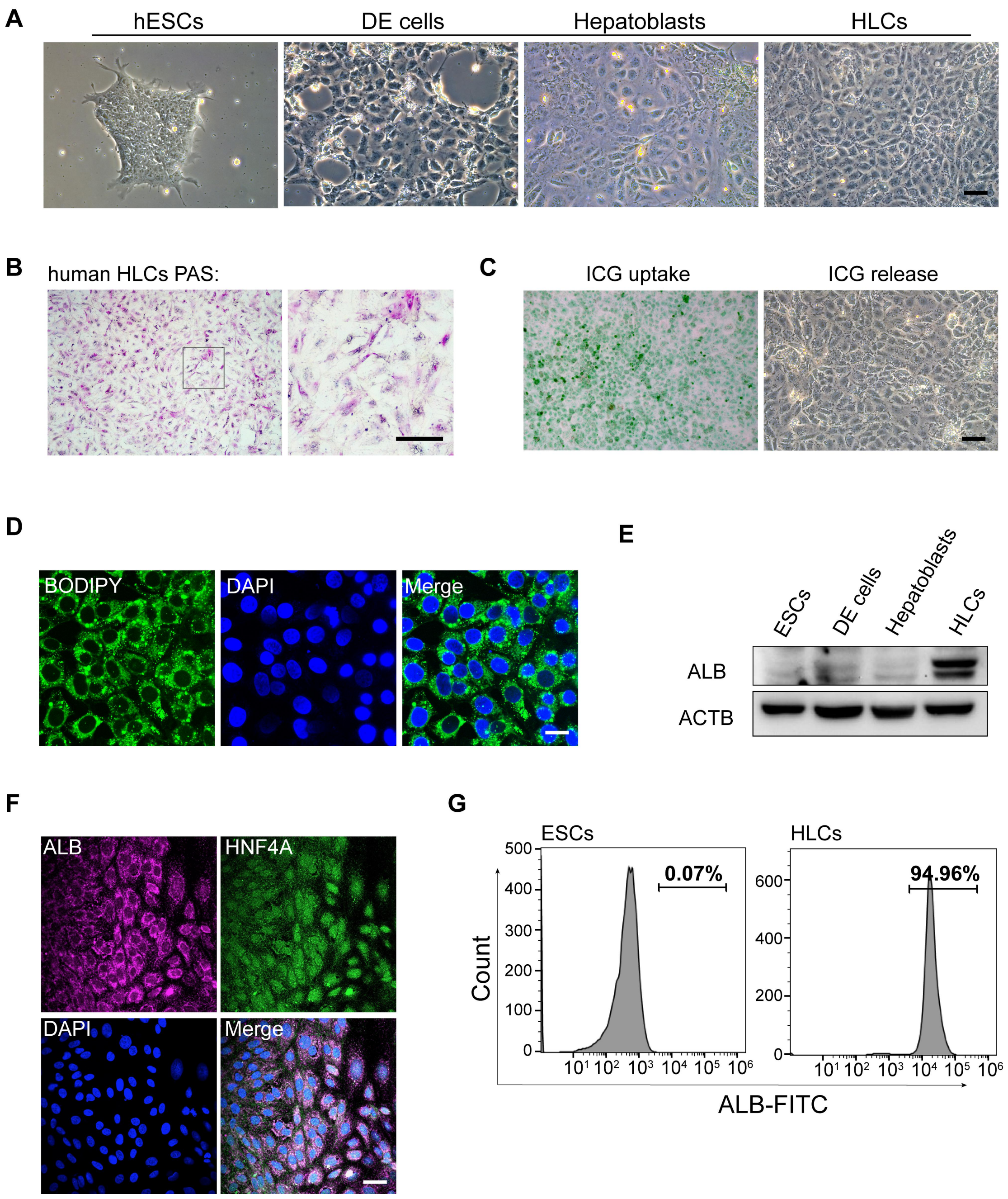
A
hESCs
DE cells
Hepatoblasts
HLCs
B
human HLCs PAS:
C
ICG uptake
ICG release
D
BODIPY
DAPI
Merge
E
ESCs
DE cells
Hepatoblasts
HLCs
ALB
ACTB
F
ALB
HNF4A
DAPI
Merge
G
ESCs
HLCs
0.07%
94.96%
Count
ALB-FITC

**Figure S5. Differentiation of human embryonic stem cells (ESCs) into HLCs.** (**A**) Representative images showing sequential cellular morphological changes during human ESC differentiation into HLCs; scale bar: 100 µm. (**B**) PAS staining of glycogen storage in human HLCs (n = 5); scale bar: 50 µm. (**C**) Analysis of ICG uptake (left) and overnight release (right, n = 5); scale bar: 100 µm. (**D**) BODIPY staining of lipid droplets in human HLCs (n = 5); scale bar: 20 µm. (**E**) Representative immunoblots of human ALB and ACTB proteins in the endpoint of HLC differentiation (n = 3). (**F**) Immunofluorescence staining of HNF4a and ALB in human HLCs (n = 3); scale bar: 50 µm. (**G**) Percentage of ALB-positive cells in human ESC and HLC cultures analyzed by flow cytometry (n = 3).

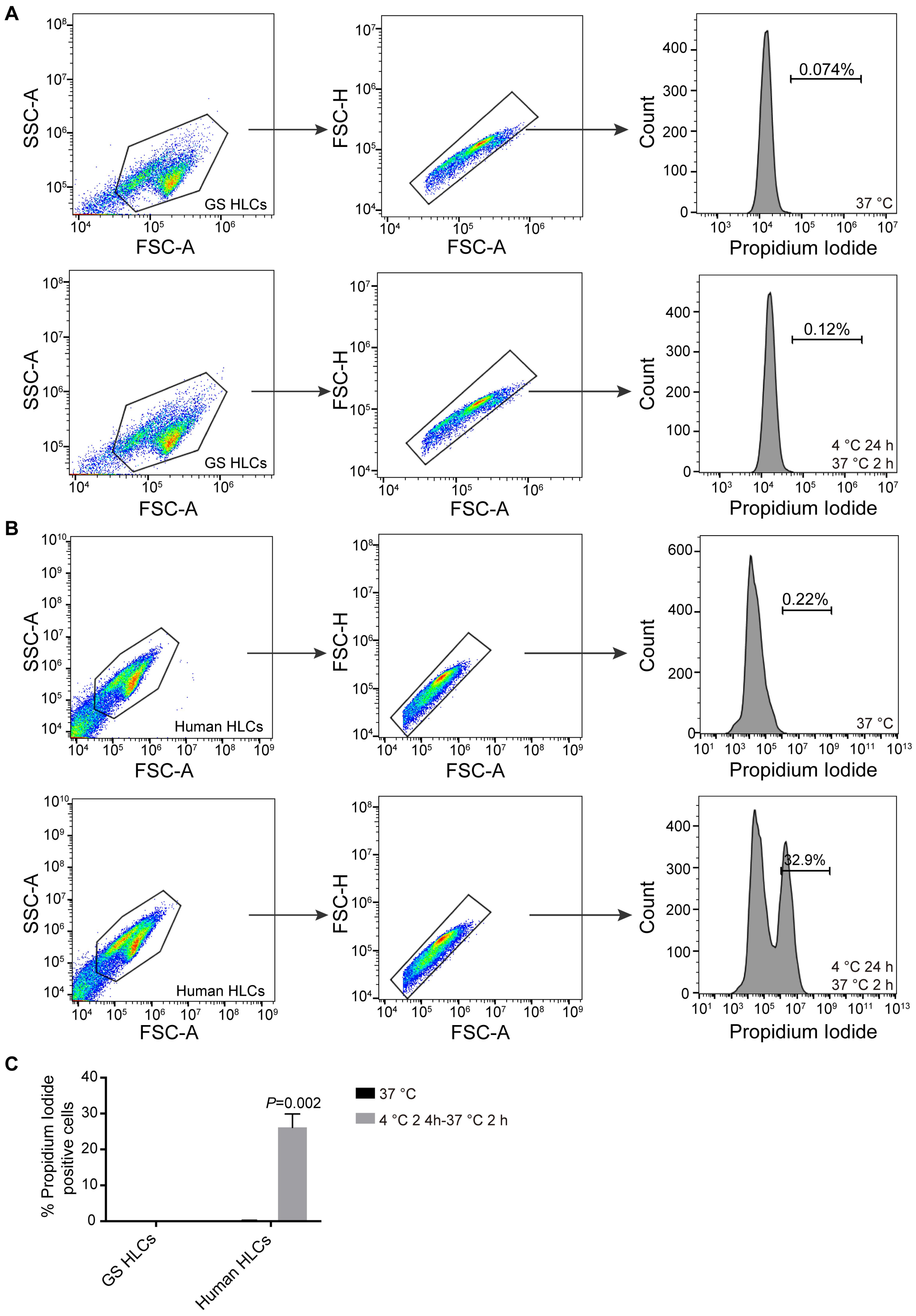
A
SSC-A
FSC-A
GS HLCs
FSC-H
Count
Propidium Iodide
0.074%
37 °C
0.12%
4 °C 24 h
37 °C 2 h
B
Human HLCs
0.22%
37 °C
32.9%
4 °C 24 h
37 °C 2 h
C
% Propidium Iodide positive cells
P=0.002
37 °C
4 °C 2 4h-37 °C 2 h
GS HLCs
Human HLCs

**Figure S6. Gating strategy of flow cytometry in characterizing propidium iodide (PI)-positive cells.** Gating strategy of PI in GS (**A**) and human (**B**) HLCs at indicated conditions analyzed by flow cytometry. (**C**) Quantification of PI-positive cells in GS and human HLCs (n = 3). Data are expressed as mean ± SEM; Student's t-test between two comparisons; *P* value is indicated.

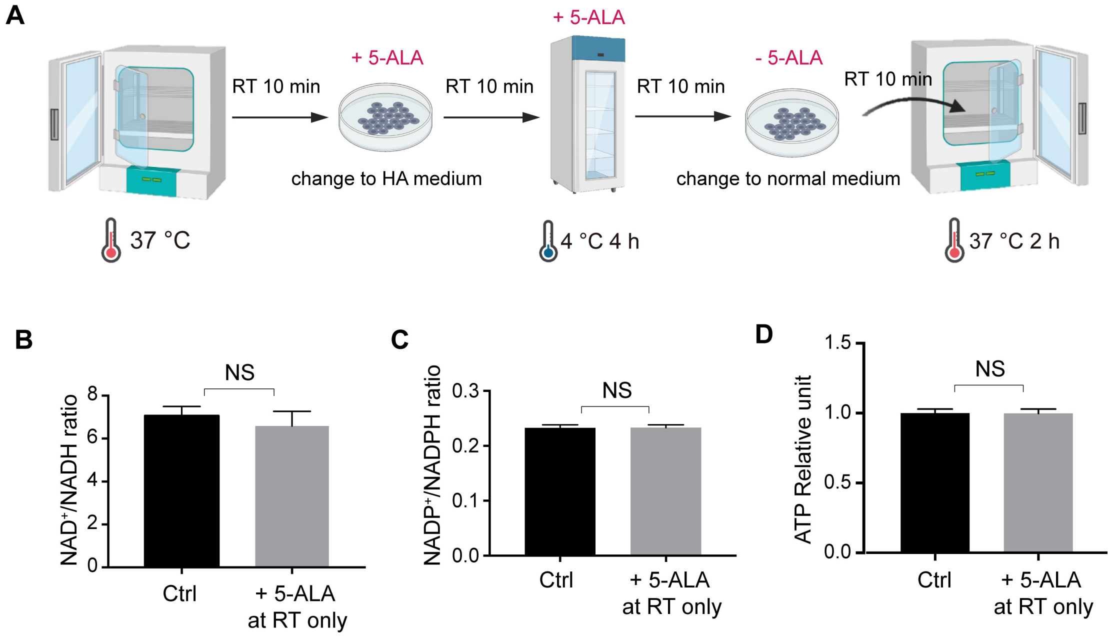
A
+ 5-ALA
RT 10 min
RT 10 min
RT 10 min
RT 10 min
- 5-ALA
change to HA medium
change to normal medium
37 °C
4 °C 4 h
37 °C 2 h
B
NAD+/NADH ratio
NS
Ctrl
+ 5-ALA at RT only
C
NADP+/NADPH ratio
NS
Ctrl
+ 5-ALA at RT only
D
ATP Relative unit
NS
Ctrl
+ 5-ALA at RT only

**Figure S7. Effects of 5-ALA supplement to human HLCs.** (**A**) Schematic diagram of cold incubation and rewarming of human HLCs with or without 5-ALA (see **Methods**). Incubation with 1 mM 5-ALA at room temperature (RT) for 10 min only showed little effect on NAD+/NADH ratio (**B**), NADP+/NADPH ratio (**C**) and ATP level (**D**) in human HLCs (n = 3). Data are expressed as mean ± SEM; Student's *t*-test between two comparisons; NS: not significant.

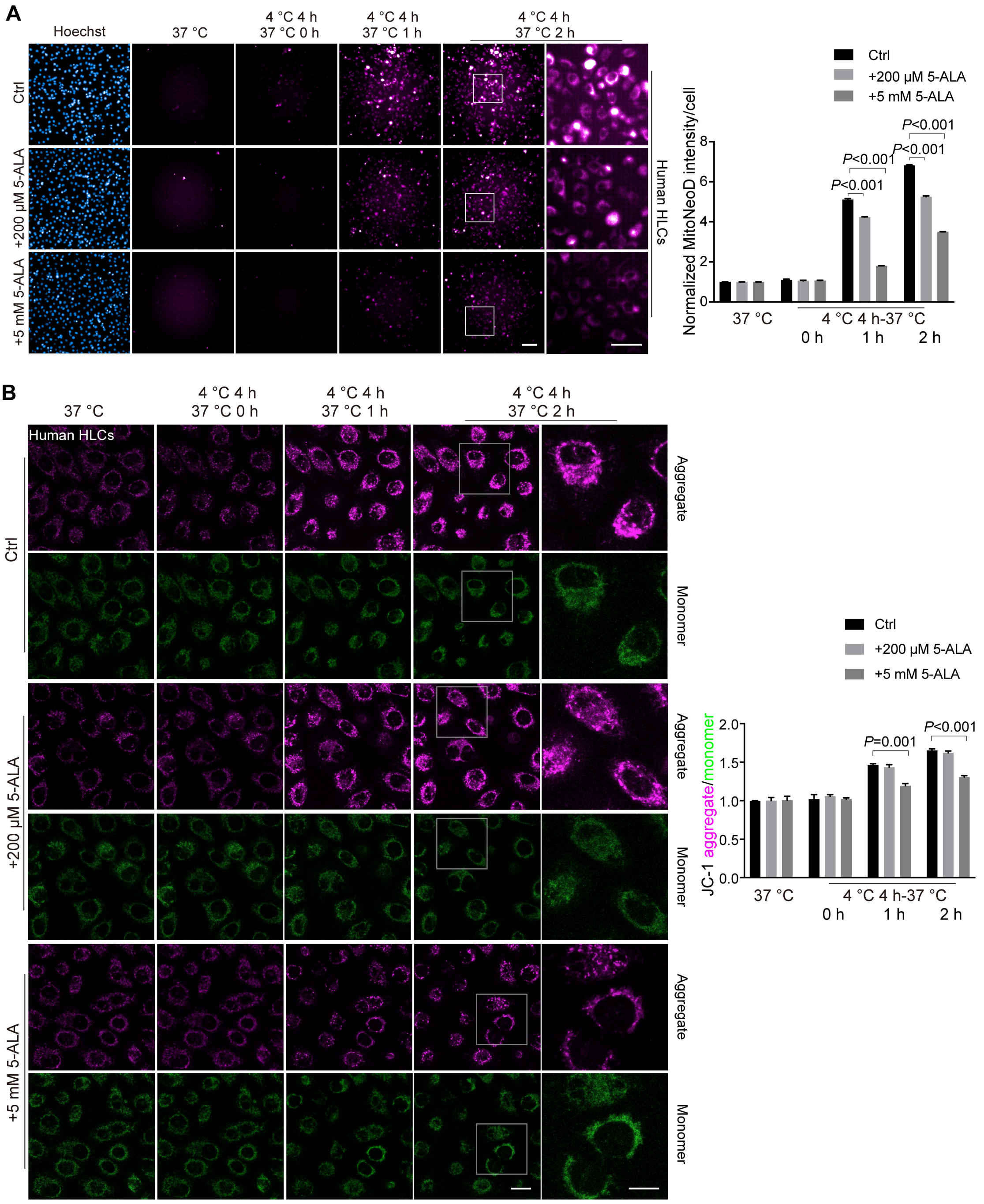
A
Hoechst
37 °C
4 °C 4 h
37 °C 0 h
4 °C 4 h
37 °C 1 h
4 °C 4 h
37 °C 2 h
Ctrl
+200 μM 5-ALA
+5 mM 5-ALA
Human HLCs
Ctrl
+200 μM 5-ALA
+5 mM 5-ALA
Normalized MitoNeoD intensity/cell
P<0.001
P<0.001
P<0.001
P<0.001
37 °C
4 °C 4 h-37 °C
0 h
1 h
2 h
B
37 °C
4 °C 4 h
37 °C 0 h
4 °C 4 h
37 °C 1 h
4 °C 4 h
37 °C 2 h
Human HLCs
Ctrl
+200 μM 5-ALA
+5 mM 5-ALA
Aggregate
Monomer
Ctrl
+200 μM 5-ALA
+5 mM 5-ALA
JC-1 aggregate/monomer
P=0.001
P<0.001
37 °C
4 °C 4 h-37 °C
0 h
1 h
2 h

**Figure S8. Dose-effects of 5-ALA treatments on mitochondrial ROS and mitochondrial membrane potential (Δψm) in human HLCs during rewarming.** (**A**) Left: automatic high-content imaging of MitoNeoD fluorescence in human HLCs during rewarming; scale bars: 100 μm; Right: quantification of MitoNeoD intensity per cell (n = 3 experiments). (**B**) Left: live imaging of JC-1 aggregate- and monomer-fluorescence to assess Δψm in human HLCs at annotated conditions during rewarming; scale bars: 20 μm; Right: quantification of JC-1 aggregate/monomer intensity (n = 3 experiments). Data are expressed as mean ± SEM; one-way ANOVA followed by Dunnett multiple-comparisons test versus ctrl group; *P* values are indicated.

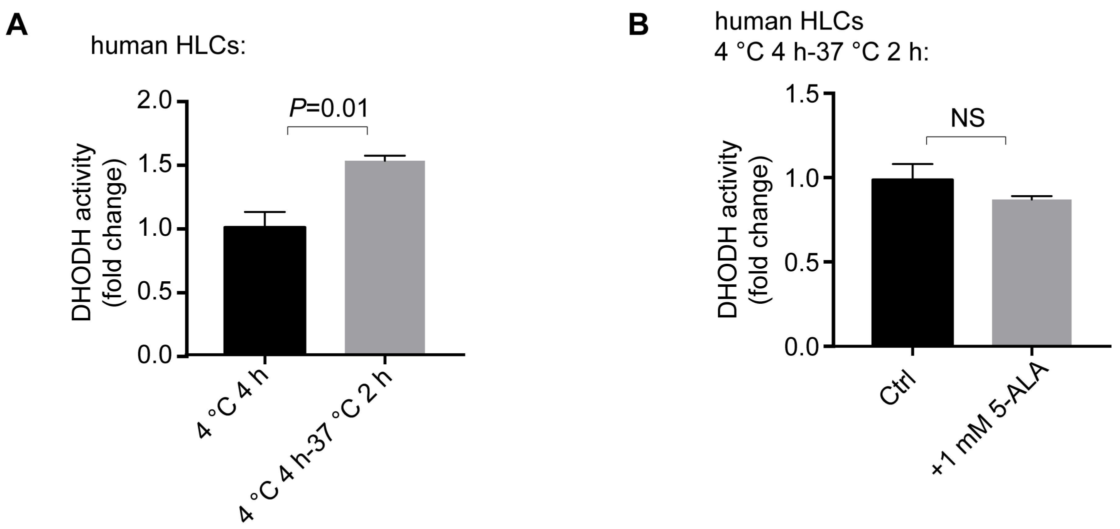
A
human HLCs:
DHODH activity (fold change)
2.0
1.5
1.0
0.5
0.0
P=0.01
4 °C 4 h
4 °C 4 h-37 °C 2 h
B
human HLCs
4 °C 4 h-37 °C 2 h:
DHODH activity (fold change)
1.5
1.0
0.5
0.0
NS
Ctrl
+1 mM 5-ALA

**Figure S9. DHODH activities in human HLCs.** (**A**) Rewarming-enhanced DHODH activity in human HLCs following 4-h cold incubation (n = 3). (**B**) Treatment with 1mM 5-ALA had no significant effect on DHODH activity in rewarmed human HLCs (n = 3). Data are expressed as mean ± SEM; Student's *t*-test between two comparisons; *P* value is indicated; NS: not significant.

**Table S1. Characteristics of human liver donors.**

| Sample number | Age | Gender | Height (cm) | Weight (kg) | BMI ($kg/m^2$) | Cause of death | Previous condition |
|---|---|---|---|---|---|---|---|
| 1 | 35 | Male | 165 | 70 | 25.7 | Stroke | Not reported |
| 2 | 11 | Male | 135 | 40 | 21.9 | Head trauma | Not reported |
| 3 | 25 | Male | 174 | 60 | 19.8 | Stroke | Not reported |
| 4 | 42 | Female | 158 | 58 | 23.2 | Stroke | Hypertension |
| 5 | 45 | Male | 173 | 75 | 25.1 | Head trauma | Not reported |
| 6 | 25 | Male | 173 | 60 | 20.0 | Head trauma | Not reported |
| 7 | 39 | Male | 165 | 60 | 22.0 | Head trauma | Not reported |
| 8 | 49 | Male | 175 | 78 | 25.5 | Stroke | Hypertension |
| 9 | 57 | Male | 170 | 68 | 23.5 | Stoke | Not reported |
| 10 | 32 | Female | 152 | 50 | 21.6 | Anoxia | Not reported |

BMI, body mass index.

**Table S2. Other reagents.**

| Reagents | Catalog # | Supplier |
|---|---|---|
| Matrigel hESC-Qualified Matrix | 354277 | Corning, USA |
| mTeSR1 Complete Kit | 85850 | Stem Cell Technologies, Canada |
| Human Hepatocyte Medium & Bullet kit (CC-3199 & CC-4182) | CC-3198 | Lonza, Switzerland |
| Fetal Bovine Serum | 1099141C | Thermo, USA |
| Hibernate-A medium | A1247501 | Thermo, USA |
| low-glucose Dulbecco's Modified Eagle Medium | C11885500BT | Thermo, USA |
| Accutase Cell Dissociation Reagent | A1110501 | Thermo, USA |
| 6-well cell culture plate | 353046 | Falcon, USA |
| RPMI 1640 Medium | 11875093 | Thermo, USA |
| B-27 Supplement(50X) | 17504044 | Thermo, USA |
| CHIR-99021 (CT99021) | S1263 | Selleck, USA |
| Y27632 | HY-10071 | MCE, USA |
| Activin A | AF-120-14E-1000 | PeproTech, USA |
| Advanced F12 | 16234010 | Thermo, USA |
| GlutaMAX Supplement | 35050061 | Thermo, USA |
| Penicillin-Streptomycin | 15140-122 | Thermo, USA |

| | | |
|---|---|---|
| Human HGF | 100-39 | PeproTech, USA |
| Oncostatin M | 300-10 | PeproTech, USA |
| Dexamethasone | D4902 | Sigma, USA |
| Paraformaldehyde | 30525-89-4 | Sigma, USA |
| Bovine serum albumin（BSA） | V900933 | Sigma, USA |
| Triton X-100 | T8787 | Sigma, USA |
| ALEXA FLUOR 488 DONKEY | A21206 | Invitrogen, USA |
| ALEXA FLUOR 594 DONKEY | A21203 | Invitrogen, USA |
| ALEXA FLUOR 647 DONKEY | A31573 | Invitrogen, USA |
| DAPI | D9542 | Sigma, USA |
| Hoechst 33342 | H3570 | Thermo, USA |
| RNeasy Mini Kit | 74104 | Qiagen, Germany |
| cDNA Reverse Transcription Kit | 11117831001 | Roche, Germany |
| AceQ Universal SYBR qPCR Master Mix | Q511-02 | Vazyme, China |
| Mammalian protein extraction Reagent | 78501 | Thermo , USA |
| Protease Inhibitor Cocktail | 87786 | Thermo , USA |
| Pierce BCA Protein Assay Kit | 23227 | Thermo , USA |
| Immobilon-P PVDF，0.45 µm | IPVH00010 | Millipore, Germany |
| Skim milk | 232100 | BD, USA |
| Immobilon Western Chemiluminescent HRP Substrate (ECL) | P90719 | Millipore, Germany |
| PAS staining kit | 3952 | Sigma, USA |
| Indocyanine Green | 1340009 | Sigma, USA |
| BODIPY 493/503 | D3922 | Thermo , USA |
| Cell Counting Kit-8 | CK04 | Dojindo, Japan |
| Propidium iodide | P4170 | Sigma, USA |
| JC-1 | C2005 | Beyotime, China |
| MitoNeoD | 563761 | Medkoo, USA |
| NAD/NADH-Glo Assay kit | G9071 | Promega, USA |
| NADP/NADPH-Glo Assay kit | G9081 | Promega, USA |
| Luciferase Assay System | E1500 | Promega, USA |
| DHO | D7128 | Sigma, USA |
| DCPIP | D1878 | Sigma, USA |
| Rotenone | R8875 | Sigma, USA |

| | | |
|---|---|---|
| Antimycin A | A8674 | Sigma, USA |
| $NaN_3$ | S2002 | Sigma, USA |
| Cytochrome c | C2867 | Sigma, USA |
| decylubiquinone | D7911 | Sigma, USA |
| Electron transport chain Complex IV activity assay kit | BC0940 | Acmec, China |
| 5-Aminolevulinic acid hydrochloride | HY-N0305 | MCE, USA |
| Krebs-Henseleit bicarbonate buffer | K3753 | Sigma, USA |
| DMEM/F12 | 36254 | Stem Cell Technologies, Canada |
| Knockout Serum Replacement | A3181502 | Thermo , USA |
| Non-Essential Amino Acids | 11140-050 | Thermo , USA |
| L-Glutamine | 25030-081 | Thermo , USA |
| L-Ascorbic acid | HY-B0166 | MCE, USA |
| b-FGF | 233-FB-025 | R&D , USA |
| University of Wisconsin solution | SPS-1 | Organ Recovery Systems, USA |
| In Situ Cell Death Detection Kit | 11684795910 | Roche, USA |
| p-dimethyl aminobenzaldehyde | 100-10-7 | Sigma, USA |
| glacial acetic acid | 64-19-7 | Sigma, USA |
| perchloric acid | 7601-90-3 | Sigma, USA |
| porphobilinogen | 487-90-1 | Sigma, USA |
| Amicon Ultra-15 Centrifugal Filter Unit | UFC9050 | Millipore, Germany |

**Table S3. Sequences of qPCR primers and siRNAs.**

| **qPCR target genes** | **5’ primer** | **3’ primer** |
|---|---|---|
| *OCT4* | CAAACGACCATCTGCCGCTTT | GGTTCTCATTGTTGTCTGCTTCCTC |
| *NANOG* | GGAGCAATCAGACCTGGAACAAC | CTCCAAGACTGGCTATTCCAAGACT |
| *SOX17* | ACCTTCACAATGCTGAGTTGAG | GGTACTTGTAGTTGGGATGGTCT |
| *GATA4* | TACGCATCTCCTGTCAGCCAGT | TGACTCTCAGCCAAGACCAGACT |
| *FOXA2* | CGCCTTCAATCATCCTTTCTCCATC | CTGTTCGTAGGTCTTGAGGTCCATT |
| *AFP* | GGCATGAAGTGAATCCTGT | TGGTGGAGGAACATAGGTC |

| | | |
|---|---|---|
| | GAACC | TCATCT |
| *HNF4A* | GGGTGTCCATTCGCATCCTTGA | GAGGCAGGCGTACTCATTGTCAT |
| *CK18* | AGAACCGAGAGGAGCTGGACAA | AAGGACTGGACTGTGCGTCTCA |
| *CK19* | GGCATGAAGTGAATCCTGTGAACC | TGGTGGAGGAACATAGGTCTCATCT |
| *TBX3* | AGGAGACAGGAACTTCGGATGAGT | AGTGGACACTGCTGGTGAGGAA |
| *ALB* | GGTGATATGGCTGACTGCTGTG | AGTGGAGGGATGGTAGGAGTATCT |
| *AAT* | CACCACTGCTCTCTTCATTCTTCCT | GCTCAGTCCACTTGTCCAAATTGTC |
| *APOA1* | CGGCATTTCTGGCAGCAAGATG | GCAGAGGTTTCAAATTGGGTCACA |
| *TTR* | GCTTCTCACCATCTACTCCTCCTC | TTCACAGACACCTCCACAGCAG |
| #1si*ALAS1* | CAGCAACGTCTTCTGCAAA | |
| #2si*ALAS1* | CCAATGACTCAACCCTCTT | |

**Table S4. Primary antibodies.**

| Antibody | Catalog # | Supplier | Concentration |
|---|---|---|---|
| SOX17 | sc-130295 | Santa Cruz | IF 1:50 WB 1:200 |
| GATA4 | sc-25310 | Santa Cruz | IF 1:50 WB 1:200 |
| HNF4a | 3113 | CST | IF 1:1000 |
| AFP | sc-51506 | Santa Cruz | IF 1:50 WB 1:200 |
| AAT | sc-59438 | Santa Cruz | IF 1:200 |
| ALB | CLF301-2 | CEDARLANE | IF 1:1000 |
| ALB | sc-271605 | Santa Cruz | WB 1:200<br>FCM 1:200 |
| OCT4 | sc-5279 | Santa Cruz | WB 1:200 |
| ACTB | 4970S | CST | WB 1:1000 |
| CYP3A4 | Sc-53850 | Santa Cruz | FCM 1:200 |
| 4-HNE | Ab46545 | Abcam | WB 1:1000 |

# Supplementary methods

## RNA sequencing

Total RNA was prepared from isolated perfused rat livers in UW solution group or UW + 5-ALA group. A total amount of 1μg RNA per liver was used as input material. The NEBNext UltraTM RNA Library Prep Kit for Illumina (NEB, USA) was used to generate cDNA libraries according to manufacturer's protocol and index codes were added to attribute sequences to each sample. The products were purified (AMpure XP system) and library quality was assessed on Bioanalyzer 2100 system (Agilent USA). Clustering of the index-coded samples was performed using TruSeq PE Cluster Kit v3-cBot-HS (Illumia, USA) on cBot Cluster Generation System. After cluster generation, the prepared libraries were sequenced on an Illumina Novaseq platform and 150 bp paired-end reads were generated.

## Quality control

Raw data (raw reads) of fastq format were firstly processed through in-house perl scripts. In this step, clean data (clean reads) were obtained by removing adapter, and excluded reads containing ploy-N and low-quality from raw data. At the same time, Q20, Q30 and GC content the clean data were calculated. All the downstream analyses were based on the clean data with high quality.

## Reads mapping to the reference genome

Reference genome and gene model annotation files were downloaded from ensemble website directly[1]. Index of the reference genome was built using Hisat2 v2.0.5 and paired-end clean reads were aligned to the reference genome using Hisat2 v2.0.5[2].

## Quantification of gene expression level

For quantification of gene expression, reads pairs were excluded that have their two ends mapping to different chromosomes or mapping to same chromosome but on different strands, and only count read pairs that have both ends aligned. Only the reads

aligned to exons, and with the mapping quality score larger than 10 were counted using the featureCounts v2.0.1 package[3].

**Differential expression analysis**

Differential expression analysis was performed by DESeq2 R package (1.26.0)[4] using a model based on the negative binomial distribution. The resulting P-values were adjusted using the Benjamini and Hochberg's approach for controlling the false discovery rate.

**Heatmap and Enrichment analysis**

Genes with the adjusted P-value < 0.05 and $log_2$Foldchange > 0.5 were considered to be significantly differentially expressed. Heatmap of genes were plotted using R, and functional enrichment analyses of differentially expressed genes were performed by the clusterProfiler R package(3.14.3)[5].

**References**

1. Zerbino DR, Achuthan P, Akanni W, Amode MR, Barrell D, Bhai J, et al. Ensembl 2018. Nucleic Acids Res. 2018; 46: D754-D61.
2. Kim D, Paggi JM, Park C, Bennett C, Salzberg SL. Graph-based genome alignment and genotyping with HISAT2 and HISAT-genotype. Nat Biotechnol. 2019; 37: 907-15.
3. Liao Y, Smyth GK, Shi W. featureCounts: an efficient general purpose program for assigning sequence reads to genomic features. Bioinformatics. 2014; 30: 923-30.
4. Love MI, Huber W, Anders S. Moderated estimation of fold change and dispersion for RNA-seq data with DESeq2. Genome Biol. 2014; 15: 550.
5. Yu G, Wang LG, Han Y, He QY. clusterProfiler: an R package for comparing biological themes among gene clusters. OMICS. 2012; 16: 284-7.
